# Chronic non-nutritive sweetener and free sugars consumption alters decision-making and risk-taking in young healthy adults

**DOI:** 10.3389/fnut.2026.1796516

**Published:** 2026-04-07

**Authors:** Camille Eustache, Sylvie Granon, Sabrina Teyssier, Nicolas Darcel

**Affiliations:** 1 AgroParisTech, INRAE, UMR PNCA, Université Paris-Saclay, Palaiseau, France; 2 CNRS, UMR Neuro-PSI, Université Paris-Saclay, Gif-sur-Yvette, France; 3 GAEL, INRAE, CNRS, Grenoble INP, Université Grenoble Alpes, Grenoble, France

**Keywords:** non-nutritive sweeteners, free sugar, decision-making, risk-taking, emotion regulation, social behavior

## Abstract

**Background:**

Non-nutritive sweeteners (NNS) are increasingly used as sugar substitutes to enhance food palatability without adding calories. Although their metabolic effects are well documented, their cognitive and socio-emotional correlates remain unclear.

**Objective:**

This study investigated the associations between chronic NNS consumption, cognitive performance, and socio-emotional behaviors in healthy young adults, controlling for free sugars intake to isolate substance-specific effects.

**Methods:**

A total of 291 French adults aged 18–30 years completed five computerized tasks assessing decision-making and emotion regulation: the Iowa Gambling Task (IGT), the Game of Dice Task (GDT), the binary dictator game, the Prisoner’s Dilemma, and the Emotion Regulation Questionnaire (ERQ). Daily NNS and free sugars intakes were estimated using quantitative food frequency questionnaires (q-FFQs). Logistic mixed-effects regression models were used to examine the effects of NNS and free sugars intake on choice probabilities.

**Results:**

Higher NNS intake tended to be associated with selecting an advantageous yet risky option relative to its non-risky counterpart during the final experiential risk block of the IGT (OR = 1.34, 95% CI [1.06, 1.69]; *p* = 0.063, Bonferroni-corrected). Under explicit risk (GDT), NNS intake was also significantly positively associated with selecting the riskiest option over the moderate risky option (OR = 1.35, 95% CI [1.12, 1.62]; *p* = 0.006, Bonferroni-corrected). Higher free sugars intake was negatively associated with selecting a disadvantageous and non-risky option relative to its risky counterpart during the final IGT block (OR = 0.78, 95% CI [0.66, 0.94]; *p* = 0.028, Bonferroni-corrected). No consistent association was observed for free sugars in the GDT. Free sugars intake was negatively associated with cognitive reappraisal (*p* = 0.027) and expressive suppression (*p* = 0.028), whereas NNS intake tended to be positively associated with cognitive reappraisal only (*p* = 0.062). Neither NNS nor free sugars intake had significant effect on altruism or cooperative behavior.

**Conclusion:**

Chronic NNS and free sugars consumption were differentially associated with decision-making under explicit and experiential risk, as well as with emotion regulation, but not prosocial behaviors. These results provide novel insights into how dietary sweeteners may influence risk-related cognitive and emotional processes in healthy young adults.

## Introduction

For decades, food manufacturers have relied on non-nutritive sweeteners (NNS)—such as aspartame, acesulfame-K, sucralose, and saccharin—as sugar substitutes that enhance food palatability without adding calories (1). Replacing sugar with NNS helps mitigate adverse health effects of excessive sugar consumption, including metabolic disorders (e.g., type 2 diabetes, cancer, cardiovascular disease) (2) and behavioral or emotional alterations such as addictive behaviors, emotional dysregulation, and cognitive decline (3–6). The global artificial sweetener market continues to grow rapidly (7), making a reduction in NNS exposure unlikely (8–10).

The metabolic effects of NNS have been extensively studied, with no consistent evidence of adverse effects on body weight or glucose regulation (11–13), and no acute alterations in postprandial metabolic or endocrine responses (14–16), their cognitive and behavioral consequences remain far less understood (17). Addressing this gap, the present study specifically examines the putative effects of NNS consumption on cognitive and socio-emotional behaviors, controlling for free sugars consumption to isolate substance-specific effects.

Complex decision-making involves uncertainty management, prosocial behavior, and emotion regulation strategies. Decision-making under uncertainty can be divided into risk (known or calculable probabilities) and ambiguity (unknown probabilities) (18). Within the domain of risk, two modalities can be distinguished: descriptive or explicit risk, in which probabilities are explicitly provided, and experiential risk, in which probabilities are learned through experience after an initial phase of ambiguity (19). Recent evidence suggests that NNS may influence decision-making under experiential risk. In adult mice, consumption of low doses of saccharin (0.012 and 0.1%) and sucralose (0.008%) administered over 5- to 6-week altered choice strategies. A higher proportion of mice exposed to 0.1% saccharin and 0.008% sucralose adopted a “risk-avoidance” and rigid choice strategy, whereas very low doses of saccharin (0.012%) increased “risk-prone” behavior, similar to sucrose, while also being associated with increased behavioral rigidity (20). However, no human study has yet examined NNS effects under uncertainty.

Beyond risk and ambiguity, this study examines prosocial behaviors, namely altruism and cooperation. Altruism involves decision with personal cost without direct benefit in return, often motivated by another’s need or distress. Cooperation, by contrast, refers to decisions whose outcomes depend on others’ actions and generally occur under strategic uncertainty (21).

Emotion regulation represents a third critical dimension. It encompasses the processes through which individuals influence the nature, intensity, timing, and expression of their emotional experiences (22). Two widely studied strategies are cognitive reappraisal and expressive suppression. Cognitive reappraisal, an antecedent-focused strategy, modifies the interpretation of emotion-eliciting situations to reduce affective impact (23). Expressive suppression, a response-focused strategy, inhibits outward emotional expressions rather than internal experience (24). Frequent reappraisal is linked to greater psychological well-being, whereas habitual suppression is associated with higher depressive symptoms (24).

To date, no study has examined NNS effects on altruism, cooperation, or emotion regulation. Existing human evidence is limited to mood-related outcomes and yields mixed results, with studies reporting either no significant effects or transient increases in depressive symptoms and irritability following an aspartame-rich diet in young adults (25–27). Overall, while NNS may influence certain aspects of decision-making such as risk-taking, their effects on prosocial behaviors and emotion regulation remain largely unexplored.

To our knowledge, this is the first human study to investigate associations between chronic NNS consumption (controlling for free sugars), cognitive performance, and socio-emotional behaviors during decision-making tasks involving risk, ambiguity, and social emotional behaviors. We hypothesized that chronic NNS intake would (1) increase risk-taking under both explicit and experiential risk, (2) reduce prosocial tendencies, including altruistic and cooperative behaviors, and (3) encourage emotion regulation strategies characterized by greater emotional suppression.

To assess these hypotheses, participants completed four incentivized decision-making tasks covering ambiguity management, risk-taking based on descriptive information or personal experience, and prosocial behaviors. The emotion regulation strategies were elicited with a questionnaire.

## Materials and methods

### Hypotheses

We formulated three hypotheses on the effects of chronic NNS consumption (controlling for free sugars) on decision-making and socio-emotional behavior.

First, chronic NNS intake may increase risk-taking under both explicit and experiential risk. As described previously in laboratory animals, low-dose saccharin consumption alters decision-making strategies during a gambling task based on experiential risk-taking, leading to increased “risk-prone” and rigid choices (20). Based on these findings, we further hypothesize that increased risky choices would also occur when probabilities are explicit.

Second, NNS intake may reduce prosocial tendencies, including altruism and cooperation.

Third, NNS intake may favor emotion regulation via greater expressive suppression. While no direct data exist on NNS effects on prosocial behavior or emotion regulation, we anticipate potential negative outcomes, analogous to those reported for sugar.

### Study population

A total of 296 healthy young French adults (18–30 years) were pre-recruited via GAEL’s Experimental Economics Platform in May 2024 and February 2025. Participants were initially selected based on the following inclusion criteria (Phase 1): age 18–30, French language proficiency of at least 7 out of 10 and basic computing skills. After cognitive testing, eligibility was confirmed through a questionnaire assessing diet, NNS and free sugars consumption (Phase 2). Participants with chronic diseases; neurological, psychiatric, or behavioral disorders; dietary restrictions for religious or personal reasons; intensive athletic training or weight-loss programs; food intolerances or allergies; or pregnancy or breastfeeding were excluded. Five participants were removed for missing data (weight or gender), leaving 291 for analysis.

The study was approved by the Research Ethics Committee of the University of Paris-Saclay (CER Paris-Saclay-2024-36), and all participants provided written informed consent.

### Assessment of cognitive function

All five decision-making tasks were developed using PsyToolkit versions 3.4.4 and 3.6 (28, 29).

#### Iowa gambling task

The Iowa Gambling Task (IGT), developed by Bechara et al. (30), was designed to detect real-life decision-making deficits, particularly those linked to ventromedial prefrontal cortex dysfunction (vmPFC). The task presents four options (A–D) with specific monetary gains and losses. At the outset, participants are unaware of outcome magnitudes and probabilities.

In our version, options A and B yield higher immediate gains (€0.50) than C and D (€0.25) but differ in loss probabilities and magnitudes: A and C involve smaller, frequent losses; B and D, larger, infrequent losses. Consequently, A and C are safer compared to B and D (see Table 1).

**Table 1:** Characteristics of the four options in the Iowa Gambling Task (IGT).

Option	Gain amount	Lost amount	Loss probability	Expected value	Category
A	€0.50	−€1.50	0.25	€0	Disadvantageous—non-risky
B	€0.50	−€3.50	0.125	€0	Disadvantageous—risky
C	€0.25	−€0.25	0.25	€0.125	Advantageous—non-risky
D	€0.25	−€0.75	0.125	€0.125	Advantageous—risky

Options A and B yield an expected gain of €0 (disadvantageous), while C and D yield €0.125 (advantageous), reflecting long-term losses vs. gains. The IGT assesses the ability to manage ambiguity—characterized by unknown outcome probabilities—in the early phase (31, 32), and risk-taking based on feedback in the later phase (32). A rational agent would favor options C and D to maximize long-term expected value.

#### Game of dice task

The Game of Dice Task (GDT) assesses risk-taking behavior under explicit and known probabilities. In this version, before each of the 18 rolls of a six-sided die, participants selected combinations of one to four sides, each associated with predefined gains or losses (€3, €1.50, €1, or €0.75, respectively; see Table 2), following the paradigm described by Brand et al. (33).

**Table 2:** Characteristics of the four choice combinations in the Game of Dice Task (GDT).

Combination	Gain amount	Lost amount	Loss probability	Expected value	Category
1-face	€3.00	−€3.00	5/6	−€2.00	Riskiest
2-face	€1.50	−€1.50	2/3	−€1.00	Moderately Risky
3-face	€1.00	−€1.00	1/2	€0	Neutral
4-face	€0.75	−€0.75	1/3	€0.25	Safest

Expected value was calculated as (Gain x Probability of winning) + (Loss x Probability of loss).

Risk level is defined by the combination of loss probability and loss magnitude (i.e., *risk = probability × severity*). Options with higher loss probabilities and larger potential losses are considered “risky,” whereas those with lower probabilities and smaller stakes are deemed “safe.” A risk-neutral, rational agent would therefore preferentially select moderate- or low-risk combinations (e.g., 3- or 4-face combinations), which constitute the optimal strategy under the task parameters.

#### Binary dictator game

The Binary Dictator Game assesses altruistic decision-making by asking participants to choose between two predefined token distributions between themselves and a charity (34). Each trial features a distinct scenario (e.g., “keep 0/donate 60” vs. “keep 48/donate 12”). Half of the trials oppose equal and unequal distributions; the other half contrast two unequal ones. Participants were informed that each token was worth €0.01. The task captures both overall and context-dependent altruism.

#### Prisoner’s dilemma

The iterated Prisoner’s Dilemma is widely used to evaluate cooperative behavior (35, 36). One the day of the experiment, participants were randomly paired with another present participant. In each trial, they chose to cooperate (Option A: share a token) or betray (Option B: keep the token). Payoffs were explicit: mutual cooperation yielded €1 each, mutual betrayal €0.50 each, and unilateral betrayal €1.50 for the betrayer and €0 for the cooperator (36).

In the first trial, participants chose without partner information. In the second, decisions were made under two hypothetical conditions: assuming the partner had cooperated or betrayed in the first trial. The task is incentive-compatible using the “strategy method” (37). For each pair, one participant was randomly selected for payment based on their first-trial decision, and the other based on their second-trial conditional decision.

#### Emotion regulation questionnaire

The Emotion Regulation Questionnaire (ERQ) assessed individual differences in cognitive reappraisal and expressive suppression. Cognitive reappraisal changes the interpretation of an emotional stimulus to reduce its impact, while expressive suppression inhibits emotion-expressive behavior after the emotion arises (22, 24).

The ERQ was adapted into French by Christophe et al. (38) to evaluate emotion regulation differences in individuals with and without anxiety. While no optimal strategy exists, cognitive reappraisal tends to yield better psychological outcomes, whereas frequent expressive suppression is less adaptive.

### Estimation of NNS and free sugars daily intake

A 24-item quantitative food frequency questionnaire (q-FFQ) estimated solid and liquid NNS intake over the past year. The frequency scale, adapted from a validated study (39), comprised seven categories (from “never or <1 time per week” to “3 times per day”). Items were tailored to products available on the French market, based on an analysis of approximately 450 products identified across major retail and online distribution channels in France. Daily intake estimates used standard serving sizes from Ciqual (40) and SU. VI. MAX (41) databases, accounting for sweetener type and relative sweetening power compared to sucrose.

The sugar q-FFQ used the same scale and included 44 items on French food products. Estimation of daily sugar intake from foods and beverages primarily contributing to free sugars, calculated as the sum of sugars from both regular and sweetened products, using the same reference databases. For dairy products, total sugars include lactose, which may lead to a slight overestimation of free sugars intake.

### Study design

Pre-selected young adults from inclusion Phase 1 were invited to complete computerized cognitive tasks in GAEL’s Experimental Economics Laboratory. In the first stage, participants performed five decision-making tasks presented in a fixed order: IGT (30), GDT (33), Binary Dictator Game (34), Prisoner’s Dilemma (36), and ERQ (24). In the second stage, anthropometric, sociodemographic, behavioral data, and NNS and free sugars intake were collected.

Participants received €10 for participation plus performance-based earnings from the first four tasks, averaging €27 total.

### Statistical analysis

Statistical analyses were conducted using R version 4.5.0 (42) within the RStudio environment (43).

### Measures

The study population was described using counts and associated percentages for categorical variables (gender, education level, student status, and physical activity), and means (± standard deviation) for quantitative variables (age, body mass index—BMI, daily NNS and free sugars intake). Daily NNS intake was the main variable of interest, with free sugars intake and gender as covariates.

Choice behavior was assessed across four computerized decision-making tasks: the IGT, the GDT, the Binary Dictator Game, and the Prisoner’s Dilemma. In the IGT, probabilities of selecting options A–D—relative to a reference option—were measured across all 100 trials and 20-trial blocks, with a rigidity score calculated for early (blocks 1–2) and late (blocks 4–5) phases, representing consistency of choices (e.g., 25% = almost random; 75% = mostly the same option) (20). In the GDT, probabilities of selecting 1–4 dice-face combinations—relative to a reference combination— were assessed across all 18 trials.

For the Binary Dictator Game and the Prisoner’s Dilemma, analyses focused on the probability of making altruistic and cooperative choice, according to the gain distribution and the scenario type, respectively.

Response times were recorded for all tasks, given that NNS consumption has been shown to significantly affect the emergence of choice strategies in adult mice (20).

Finally, cognitive reappraisal and expressive suppression scores from the ERQ were measured.

### Models

To examine the link between daily NNS intake and decision-making, multinomial logistic regression models with participant-level random intercepts were fitted for the IGT and GDT. Binary logistic regression models with random intercepts were used for the Binary Dictator Game and the Prisoner’s Dilemma, modeling the probability of altruistic and cooperative choices. Daily free sugars intake, age, gender, BMI, education level and physical activity were included as covariates, and continuous predictors were standardized (z-scores) prior to analysis. Free sugars and NNS intake were first log-transformed to reduce right-skewness and then standardized. Student status was excluded due to its strong correlation with education level / most then 80% of participants are student, simplifying the analysis.

For example, the multinomial logistic regression for the IGT was specified as follows:


$$\begin{array}{l} \text{logit}(P(Y_{\mathrm{ij}}=k))=\\ \beta_{0}+\beta_{1}\mathrm{NN}S_{i}+\beta_{2}\text{Suga}r_{i}+\beta_{3}\mathrm{Ag}e_{i}+\beta_{4}\text{Gende}r_{i}+\text{βnCovariat}e_{i}+u_{i} \end{array}$$

where $Y_{\mathrm{ij}}$ represents the choice of option k by participant i on trial j , $u_{i}\;$ is the random intercept for participant i , NNS denotes daily intake of non-nutritive sweeteners; Sugar denotes daily free sugars intake; Age and Gender are participant-level variables; and Covariate represents additional covariates included in the model.

*p*-values were adjusted for multiple comparisons using the Bonferroni correction, with NNS intake treated as the primary exposure of interest. Given their conceptual and methodological relevance to this exposure, free sugars intake (as a control variable) age, and gender were also included in the correction procedure. In multinomial models, the correction was applied separately within each equation, whereas in binomial models it was applied across these pre-specified primary predictors only. Other covariates were included as adjustment variables and were not part of the multiple-testing correction procedure.

To explore overall differences in choice behavior, independent of explanatory variables, additional multinomial and binary logistic models including only random intercepts were fitted for all for tasks.

Response times were log-transformed prior to analysis due to strong positive skewness. Log-transformed response times were analyzed using linear mixed-effects models, incorporating interaction terms between option type and trial or block when relevant. Bonferroni correction was applied for multiple comparisons. Another linear mixed-effects model assessed the association between NNS intake and rigidity scores across task phases (early vs. late blocks), controlling for free sugars intake, gender, BMI, education level and physical activity. Bonferroni correction was similarly applied for IGT rigidity scores. Finally, ANCOVA models without interactions were used to evaluate the relationship between daily NNS intake and emotion regulation indices (cognitive reappraisal and expressive suppression) adjusting for the same covariates. Bonferroni correction was not applied to these models, as only one test per score was performed.

### Sample size

The sample size was determined via power analysis, based on one of the primary outcome measures of the study: the IGT. Expected means (∆ = 10.61), standard deviations (SD = 25.18), and alpha levels (*p* < 0.05) were taken from prior IGT studies (44–51). A minimum of 90 participants per group (no/low, average, and high consumption of sweeteners) was required for 80% statistical power. To meet this requirement, approximately 370 young adults were initially preselected. However, the average- and high-consumption groups—especially the high group (42 participants)—fell short of the target. Therefore, logistic regression analyses were conducted using daily NNS intake as a continuous variable instead of predefined groups.

## Results

### Participants characteristics

A total of 291 young adults were included in the statistical analyses. The mean age of participants was 22.01 ± 2.95 years, with a nearly equal gender distribution (47.1% Men, 52.9% Women). The average body mass index (BMI) was 22.73 ± 3.73 kg.m^−2^. Most participants were students (83.5%), and 60.14% had an education higher than a 2-year post-baccalaureate. Physical activity levels were distributed as follows: 30.9% low, 36.4% moderate, and 32.7% high. The mean daily intake of free sugars was 101.0 ± 85.4 g, while the mean intake of NNS was 30.8 ± 83.3 mg.

### Iowa gambling task

#### Probability of selecting an option

**Figure 1:**
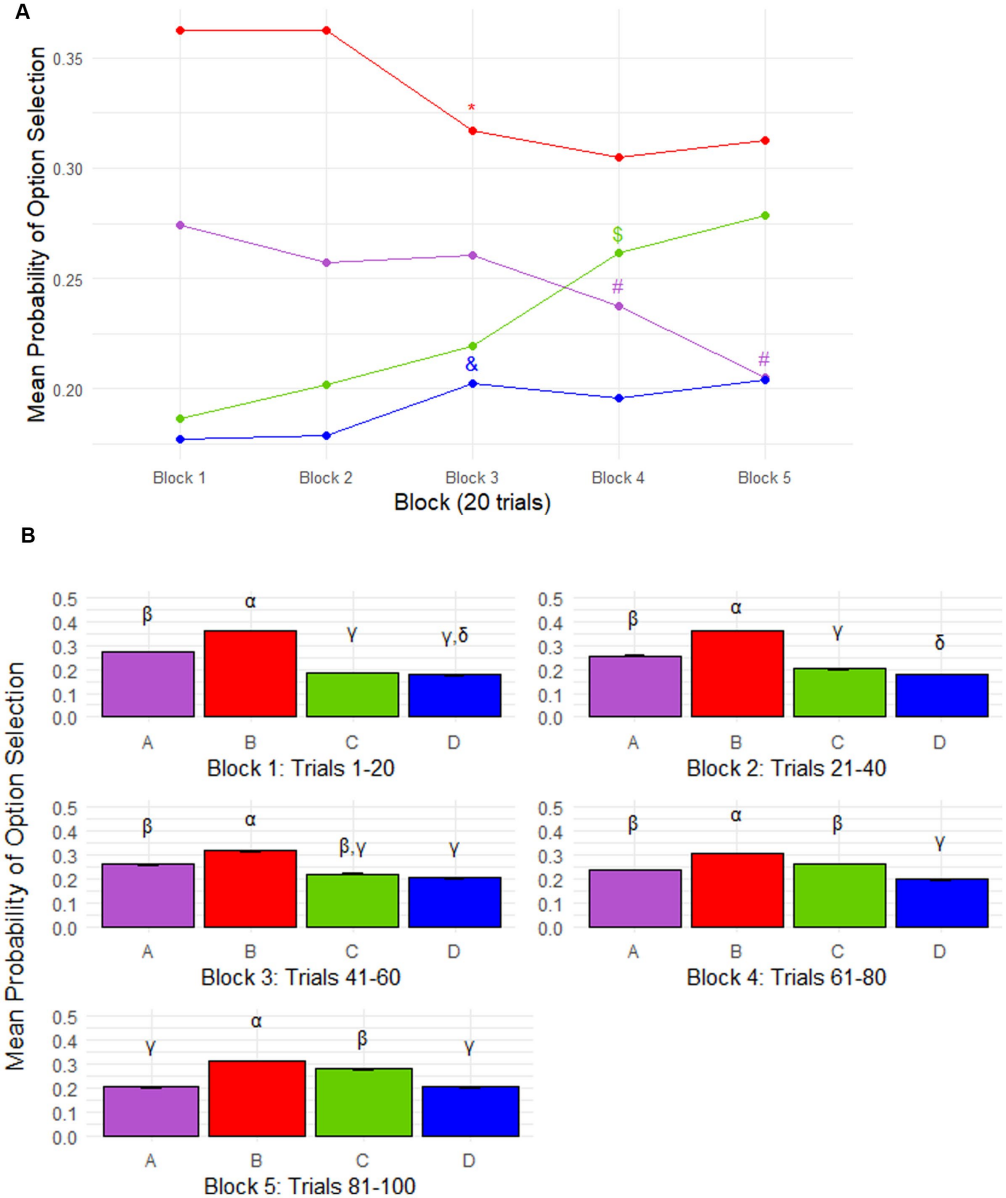
Iowa gambling task (IGT) performance. **(A)** Mean probability of option selection across 20-trial IGT blocks for all participants. Selection probabilities changed across blocks, reflecting the learning phase. Blocks are defined as follows: Block 1 = trials 1–20, Block 2 = trials 21–40, Block 3 = trials 41–60, Block 4 = trials 61–80, Block 5 = trials 81–100. Between blocks 2 and 3, the probability of selecting option B decreased significantly (*p* < 0.0001), while option D increased (*p* = 0.007); both then stabilized over the remaining 40 trials. Option A decreased progressively from block 3 (*p* = 0.026 and *p* = 0.0001 for blocks 3–4 and 4–5, respectively), whereas option C increased from block 2, with significant rises between Blocks 3–4 (*p* < 0.0001), then stabilized. Options are color-coded as follows: A (purple), B (red), C (green), D (blue). Symbols indicate significant changes from the previous block (*p* < 0.05): * = B, **#** = A, $ = C, & = D. **(B)** Mean probability of option selection within each 20-trial IGT block for all participants. During the learning phase (Blocks 1–2; trials 1–40), the following pattern emerged: B > A > C > D. In block 3, no significant difference was found between selection probabilities of options D and C (*p* = 0.131), nor between C and A in block 4 (*p* = 0.563). By the final block (Block 5), selection of option C was significantly higher than option A (*p* = 0.015), with no significant difference between D and A (*p* = 0.612). Overall, by the end of the task, the choice pattern was B > C > A = D. Different Greek letters indicate significant differences between options (*p* < 0.05).

The mean probability of selecting option A, B, C, or D differs significantly across the 100 trials. The probability of selecting option B (p(B) = 0.33 ± 0.14; *p* < 10^−9^) was significantly the highest, whereas the probability of selecting option D (p(D) = 0.19 ± 0.13; *p* = 0.003) was the lowest. The probability of selecting option C (p(C) = 0.23 ± 0.15) tended to be lower than the probability of selecting option A (p(A) = 0.25 ± 0.13; *p* = 0.095; Supplementary Figure S1). Mean selection probabilities per block are shown in Figures 1A,B.

Result 1. During the first 60 trials (Blocks 1–3), daily NNS and free sugars intake were not associated with option selection (Tables 3–5). From Block 4 onward, associations emerged.

**Table 3:** Effect of predictors on option selection in block 1 (trials 1–20) of the IGT.

Predictor: A vs. B^1^—Block 1	OR [95% CI]^2^	*p*-value	*p*-value Bonferroni^3^
Sweeteners (log-standardized)	0.97 [0.85, 1.10]	0.620	1.000
Free sugars (log-standardized)	1.00 [0.87, 1.14]	0.959	1.000
Age (standardized)	0.96 [0.84, 1.09]	0.494	1.000
Gender (Women vs. Men)	0.94 [0.74, 1.19]	0.595	1.000
Predictor: C vs. B—Block 1			
Sweeteners (log-standardized)	0.94 [0.82, 1.08]	0.407	1.000
Free sugars (log-standardized)	1.00 [0.87, 1.15]	0.998	1.000
Age (standardized)	0.96 [0.84, 1.10]	0.573	1.000
Gender (Women vs. Men)	0.98 [0.77, 1.26]	0.895	1.000
Predictor: D vs. B—Block 1			
Sweeteners (log-standardized)	1.03 [0.90, 1.19]	0.654	1.000
Free sugars (log-standardized)	1.07 [0.93, 1.23]	0.352	1.000
Age (standardized)	1.00 [0.88, 1.15]	0.973	1.000
Gender (Women vs. Men)	1.13 [0.88, 1.46]	0.331	1.000
Predictor: C vs. A^1^—Block 1			
Sweeteners (log-standardized)	0.97 [0.86, 1.10]	0.668	1.000
Free sugars (log-standardized)	1.01 [0.88, 1.14]	0.933	1.000
Age (standardized)	1.00 [0.88, 1.14]	0.955	1.000
Gender (Women vs. Men)	1.05 [0.83, 1.33]	0.670	1.000
Predictor: D vs. A—Block 1			
Sweeteners (log-standardized)	1.06 [0.93, 1.21]	0.367	1.000
Free sugars (log-standardized)	1.08 [0.94, 1.23]	0.285	1.000
Age (standardized)	1.05 [0.92, 1.19]	0.468	1.000
Gender (Women vs. Men)	1.21 [0.95, 1.54]	0.119	0.475
Predictor: D vs. C^1^—Block 1			
Sweeteners (log-standardized)	1.10 [0.97, 1.24]	0.150	0.600
Free sugars (log-standardized)	1.07 [0.95, 1.22]	0.262	1.000
Age (standardized)	1.03 [0.92, 1.17]	0.575	1.000
Gender (Women vs. Men)	1.18 [0.95, 1.48]	0.140	0.560

1 Comparisons are made between each option and its respective reference within Block 1 (trials 1–20): A, C, and D vs. B; C and D vs. A; and D vs. C, as indicated in the table.

2 Odds ratios (OR) are exponentiated coefficients from the multinomial logistic model adjusted for covariates. 95% CI: 95% confidence interval.

3 *p*-values were adjusted using the Bonferroni correction, applied separately within each equation and only to the primary predictors of interest: NNS intake, free sugars intake, age, and gender. *p* < 0.05 indicates statistical significance.

**Table 4:** Effect of predictors on option selection in block 2 (trials 21–40) of the IGT.

Predictor: A vs. B^1^—Block 2	OR [95% CI]^2^	*p*-value	*p*-value Bonferroni^3^
Sweeteners (log-standardized)	0.97 [0.84, 1.13]	0.703	1.000
Free sugars (log-standardized)	0.88 [0.76, 1.03]	0.110	0.438
Age (standardized)	0.89 [0.77, 1.04]	0.148	0.592
Gender (Women vs. Men)	0.79 [0.60, 1.03]	0.086	0.343
Predictor: C vs. B—Block 2			
Sweeteners (log-standardized)	0.93 [0.79, 1.11]	0.436	1.000
Free sugars (log-standardized)	0.98 [0.83, 1.17]	0.860	1.000
Age (standardized)	0.95 [0.80, 1.12]	0.516	1.000
Gender (Women vs. Men)	1.07 [0.78, 1.45]	0.683	1.000
Predictor: D vs. B—Block 2			
Sweeteners (log-standardized)	1.08 [0.91, 1.29]	0.371	1.000
Free sugars (log-standardized)	1.03 [0.87, 1.23]	0.718	1.000
Age (standardized)	1.03 [0.87, 1.22]	0.697	1.000
Gender (Women vs. Men)	1.15 [0.84, 1.57]	0.378	1.000
Predictor: C vs. A^1^—Block 2			
Sweeteners (log-standardized)	0.96 [0.81, 1.14]	0.647	1.000
Free sugars (log-standardized)	1.12 [0.94, 1.32]	0.211	0.844
Age (standardized)	1.06 [0.89, 1.25]	0.523	1.000
Gender (Women vs. Men)	1.37 [1.01, 1.86]	0.046*	0.182
Predictor: D vs. A—Block 2			
Sweeteners (log-standardized)	1.11 [0.93, 1.32]	0.231	0.925
Free sugars (log-standardized)	1.17 [0.98, 1.39]	0.079	0.317
Age (standardized)	1.15 [0.97, 1.37]	0.098	0.390
Gender (Women vs. Men)	1.45 [1.06, 1.98]	0.021*	0.085
Predictor: D vs. C^1^—Block 2			
Sweeteners (log-standardized)	1.16 [0.99, 1.35]	0.074	0.296
Free sugars (log-standardized)	1.05 [0.89, 1.23]	0.568	1.000
Age (standardized)	1.09 [0.93, 1.27]	0.271	1.000
Gender (Women vs. Men)	1.07 [0.80, 1.43]	0.635	1.000

1 Comparisons are made between each option and its respective reference within Block 2 (trials 21–40): A, C, and D vs. B; C and D vs. A; and D vs. C, as indicated in the table.

2 Odds ratios (OR) are exponentiated coefficients from the multinomial logistic model adjusted for covariates. 95% CI: 95% confidence interval.

3 *p*-values were adjusted using the Bonferroni correction, applied separately within each equation and only to the primary predictors of interest: NNS intake, free sugars intake, age, and gender. Bold values and “*” indicate statistically significant differences (*p* < 0.05).

**Table 5:** Effect of predictors on option selection in block 3 (trials 41–60) of the IGT.

Predictor: A vs. B^1^—Block 3	OR [95% CI]^2^	*p*-value	*p*-value Bonferroni^3^
Sweeteners (log-standardized)	0.94 [0.80, 1.12]	0.499	1.000
Free sugars (log-standardized)	0.91 [0.76, 1.08]	0.260	1.000
Age (standardized)	1.04 [0.88, 1.23]	0.660	1.000
Gender (Women vs. Men)	0.78 [0.57, 1.06]	0.108	0.434
Predictor: C vs. B—Block 3			
Sweeteners (log-standardized)	0.89 [0.73, 1.08]	0.232	0.927
Free sugars (log-standardized)	1.08 [0.88, 1.31]	0.471	1.000
Age (standardized)	0.95 [0.78, 1.15]	0.586	1.000
Gender (Women vs. Men)	0.91 [0.64, 1.30]	0.594	1.000
Predictor: D vs. B—Block 3			
Sweeteners (log-standardized)	1.06 [0.87, 1.28]	0.577	1.000
Free sugars (log-standardized)	1.08 [0.89, 1.31]	0.426	1.000
Age (standardized)	0.95 [0.79, 1.14]	0.582	1.000
Gender (Women vs. Men)	0.80 [0.57, 1.12]	0.195	0.780
Predictor: C vs. A^1^—Block 3			
Sweeteners (log-standardized)	0.94 [0.76, 1.15]	0.526	1.000
Free sugars (log-standardized)	1.19 [0.97, 1.46]	0.104	0.415
Age (standardized)	0.92 [0.75, 1.12]	0.399	1.000
Gender (Women vs. Men)	1.18 [0.82, 1.70]	0.383	1.000
Predictor: D vs. A—Block 3			
Sweeteners (log-standardized)	1.12 [0.90, 1.38]	0.308	1.000
Free sugars (log-standardized)	1.18 [0.96, 1.47]	0.119	0.477
Age (standardized)	0.92 [0.75, 1.14]	0.460	1.000
Gender (Women vs. Men)	1.02 [0.69, 1.49]	0.929	1.000
Predictor: D vs. C^1^—Block 3			
Sweeteners (log-standardized)	1.19 [1.00, 1.43]	0.056	0.222
Free sugars (log-standardized)	1.00 [0.84, 1.20]	0.974	1.000
Age (standardized)	1.00 [0.84, 1.20]	0.966	1.000
Gender (Women vs. Men)	0.88 [0.64, 1.22]	0.444	1.000

1 Comparisons are made between each option and its respective reference within Block 3 (trials 41–60): A, C, and D vs. B; C and D vs. A; and D vs. C, as indicated in the table.

2 Odds ratios (OR) are exponentiated coefficients from the multinomial logistic model adjusted for covariates. 95% CI, 95% confidence interval.

3 *p*-values were adjusted using the Bonferroni correction, applied separately within each equation and only to the primary predictors of interest: NNS intake, free sugars intake, age, and gender. *p* < 0.05 indicates statistical significance.

Daily NNS intake was not associated with option selection in Block 4 (Table 6), but it tended to be positively associated with the probability of selecting option D over option C in Block 5 (OR = 1.34, 95% CI [1.06–1.69]; *p* = 0.063, Bonferroni-corrected; Table 7) and across all trials (OR = 1.20, 95% CI [1.03–1.39]; *p* = 0.067, Bonferroni-corrected; Table 8).

**Table 6:** Effect of predictors on option selection in block 4 (trials 61–80) of the IGT.

Predictor: A vs. B^1^—Block 4	OR [95% CI]^2^	*p*-value	*p*-value Bonferroni^3^
Sweeteners (log-standardized)	0.93 [0.79, 1.09]	0.362	1.000
Free sugars (log-standardized)	0.84 [0.71, 0.99]	0.036*	0.145
Age (standardized)	0.99 [0.85, 1.17]	0.937	1.000
Gender (Women vs. Men)	0.67 [0.50, 0.90]	0.008**	0.030*
Predictor: C vs. B—Block 4			
Sweeteners (log-standardized)	0.94 [0.76, 1.16]	0.574	1.000
Free sugars (log-standardized)	0.98 [0.79, 1.21]	0.839	1.000
Age (standardized)	1.02 [0.83, 1.25]	0.868	1.000
Gender (Women vs. Men)	0.84 [0.57, 1.23]	0.373	1.000
Predictor: D vs. B—Block 4			
Sweeteners (log-standardized)	1.16 [0.96, 1.41]	0.128	0.512
Free sugars (log-standardized)	0.93 [0.76, 1.13]	0.448	1.000
Age (standardized)	1.03 [0.85, 1.24]	0.785	1.000
Gender (Women vs. Men)	0.81 [0.57, 1.15]	0.237	0.949
Predictor: C vs. A^1^—Block 4			
Sweeteners (log-standardized)	1.01 [0.81, 1.28]	0.901	1.000
Free sugars (log-standardized)	1.16 [0.92, 1.46]	0.210	0.838
Age (standardized)	1.03 [0.82, 1.29]	0.808	1.000
Gender (Women vs. Men)	1.25 [0.83, 1.90]	0.286	1.000
Predictor: D vs. A—Block 4			
Sweeteners (log-standardized)	1.25 [1.01, 1.56]	0.045*	0.179
Free sugars (log-standardized)	1.09 [0.87, 1.36]	0.456	1.000
Age (standardized)	1.04 [0.84, 1.29]	0.698	1.000
Gender (Women vs. Men)	1.18 [0.80, 1.76]	0.405	1.000
Predictor: D vs. C^1^—Block 4			
Sweeteners (log-standardized)	1.24 [0.99, 1.54]	0.057	0.230
Free sugars (log-standardized)	0.95 [0.76, 1.18]	0.628	1.000
Age (standardized)	1.01 [0.82, 1.25]	0.918	1.000
Gender (Women vs. Men)	0.96 [0.65, 1.42]	0.839	1.000

1 Comparisons are made between each option and its respective reference within Block 4 (trials 61–80): A, C, and D vs. B; C and D vs. A; and D vs. C, as indicated in the table.

2 Odds ratios (OR) are exponentiated coefficients from the multinomial logistic model adjusted for covariates. 95% CI: 95% confidence interval.

3 *p*-values were adjusted using the Bonferroni correction, applied separately within each equation and only to the primary predictors of interest: NNS intake, free sugars intake, age, and gender. Bold values and “*” indicate statistically significant differences (*p* < 0.05).

**Table 7:** Effect of predictors on option selection in block 5 (trials 81–100) of the IGT.

Predictor: A vs. B^1^—5	OR [95% CI]^2^	*p*-value	*p*-value Bonferroni^3^
Sweeteners (log-standardized)	0.97 [0.81, 1.15]	0.697	1.000
Free sugars (log-standardized)	0.78 [0.66, 0.94]	0.007**	0.028*
Age (standardized)	1.01 [0.84, 1.20]	0.942	1.000
Gender (Women vs. Men)	0.69 [0.50, 0.95]	0.024*	0.094
Predictor: C vs. B—5			
Sweeteners (log-standardized)	0.78 [0.62, 0.99]	0.042*	0.168
Free sugars (log-standardized)	1.06 [0.83, 1.34]	0.663	1.000
Age (standardized)	1.09 [0.86, 1.38]	0.478	1.000
Gender (Women vs. Men)	0.61 [0.40, 0.95]	0.028*	0.111
Predictor: D vs. B—5			
Sweeteners (log-standardized)	1.05 [0.86, 1.29]	0.648	1.000
Free sugars (log-standardized)	1.01 [0.82, 1.23]	0.950	1.000
Age (standardized)	1.10 [0.90, 1.34]	0.367	1.000
Gender (Women vs. Men)	0.75 [0.52, 1.07]	0.115	0.458
Predictor: C vs. A^1^—5			
Sweeteners (log-standardized)	0.81 [0.64, 1.03]	0.090	0.358
Free sugars (log-standardized)	1.33 [1.04, 1.70]	0.022*	0.088
Age (standardized)	1.08 [0.85, 1.37]	0.541	1.000
Gender (Women vs. Men)	0.90 [0.58, 1.39]	0.632	1.000
Predictor: D vs. A—5			
Sweeteners (log-standardized)	1.09 [0.88, 1.34]	0.434	1.000
Free sugars (log-standardized)	1.26 [1.02, 1.56]	0.029*	0.115
Age (standardized)	1.08 [0.88, 1.33]	0.437	1.000
Gender (Women vs. Men)	1.07 [0.73, 1.57]	0.714	1.000
Predictor: D vs. C^1^—5			
Sweeteners (log-standardized)	1.34 [1.06, 1.69]	0.016*	0.063
Free sugars (log-standardized)	0.96 [0.76, 1.22]	0.736	1.000
Age (standardized)	1.01 [0.80, 1.27]	0.941	1.000
Gender (Women vs. Men)	1.21 [0.79, 1.86]	0.381	1.000

1 Comparisons are made between each option and its respective reference within Block 5 (trials 81–100): A, C, and D vs. B; C and D vs. A; and D vs. C, as indicated in the table.

2 Odds ratios (OR) are exponentiated coefficients from the multinomial logistic model adjusted for covariates. 95% CI, 95% confidence interval.

3 *p*-values were adjusted using the Bonferroni correction, applied separately within each equation and only to the primary predictors of interest: NNS intake, free sugars intake, age, and gender. Bold values and “*” indicate statistically significant differences (*p* < 0.05).

Daily free sugars intake showed a progressive negative association with selecting option A over B, emerging as a trend in Block 4 (OR = 0.84, 95% CI [0.71–0.99]; *p* = 0.145, Bonferroni-corrected; Table 7), becoming significant in Block 5 (OR = 0.78, 95% CI [0.66–0.94]; *p* = 0.028, Bonferroni-corrected; Table 8), and showing a similar tendency across all trials (OR = 0.88, 95% CI [0.78–0.99]; *p* = 0.112, Bonferroni-corrected; Table 3). At the end of the task, free sugars intake also tended to be positively associated with selecting options C and D over option A (OR = 1.33, 95% CI [1.04–1.70]; *p* = 0.088; OR = 1.26, 95% CI [1.02–1.56]; *p* = 0.115, Bonferroni-corrected; Table 8). Across all trials, women selected option A significantly less often than option B and less frequently than men (OR = 0.76, 95% CI [0.62–0.94]; *p* = 0.044, Bonferroni-corrected; Table 3), a pattern emerging from Block 4 onward. Age had no significant effect on IGT choice.

**Table 8:** Effect of predictors on option selection across all 100 trials of the IGT.

Predictor: A vs. B^1^	OR [95% CI]^2^	*p*-value	*p*-value Bonferroni^3^
Sweeteners (log-standardized)	0.96 [0.86, 1.08]	0.475	1.000
Free sugars (log-standardized)	0.88 [0.78, 0.99]	0.028*	0.112
Age (standardized)	0.97 [0.86, 1.08]	0.546	1.000
Gender (Women vs. Men)	0.76 [0.62, 0.94]	0.011*	0.044*
Predictor: C vs. B			
Sweeteners (log-standardized)	0.91 [0.78, 1.05]	0.209	0.836
Free sugars (log-standardized)	1.00 [0.86, 1.16]	0.971	1.000
Age (standardized)	0.97 [0.84, 1.13]	0.723	1.000
Gender (Women vs. Men)	0.88 [0.67, 1.15]	0.348	1.000
Predictor: D vs. B			
Sweeteners (log-standardized)	1.09 [0.95, 1.24]	0.213	0.853
Free sugars (log-standardized)	1.01 [0.88, 1.15]	0.894	1.000
Age (standardized)	1.01 [0.89, 1.15]	0.858	1.000
Gender (Women vs. Men)	0.91 [0.71, 1.15]	0.421	1.000
Predictor: C vs. A^1^			
Sweeteners (log-standardized)	0.95 [0.82, 1.10]	0.478	1.000
Free sugars (log-standardized)	1.14 [0.98, 1.32]	0.096	0.383
Age (standardized)	1.01 [0.87, 1.17]	0.913	1.000
Gender (Women vs. Men)	1.15 [0.88, 1.51]	0.305	1.000
Predictor: D vs. A			
Sweeteners (log-standardized)	1.13 [0.98, 1.32]	0.100	0.400
Free sugars (log-standardized)	1.15 [0.99, 1.34]	0.071	0.285
Age (standardized)	1.05 [0.90, 1.22]	0.529	1.000
Gender (Women vs. Men)	1.19 [0.90, 1.56]	0.220	0.881
Predictor: D vs. C^1^			
Sweeteners (log-standardized)	1.20 [1.03, 1.39]	0.017*	0.067
Free sugars (log-standardized)	1.01 [0.87, 1.18]	0.867	1.000
Age (standardized)	1.04 [0.90, 1.20]	0.596	1.000
Gender (Women vs. Men)	1.03 [0.79, 1.35]	0.819	1.000

1 Comparisons are made between each option and its respective reference: A, C, and D vs. B; C and D vs. A; and D vs. C, as indicated in the table.

2 Odds ratios (OR) are exponentiated coefficients from the multinomial logistic model adjusted for covariates. 95% CI, 95% confidence interval.

3 *p*-values were adjusted using the Bonferroni correction, applied separately within each equation and only to the primary predictors of interest: NNS intake, free sugars intake, age, and gender. Bold values and “*” indicate statistically significant differences (*p* < 0.05).

#### Rigidity score

**Figure 2:**
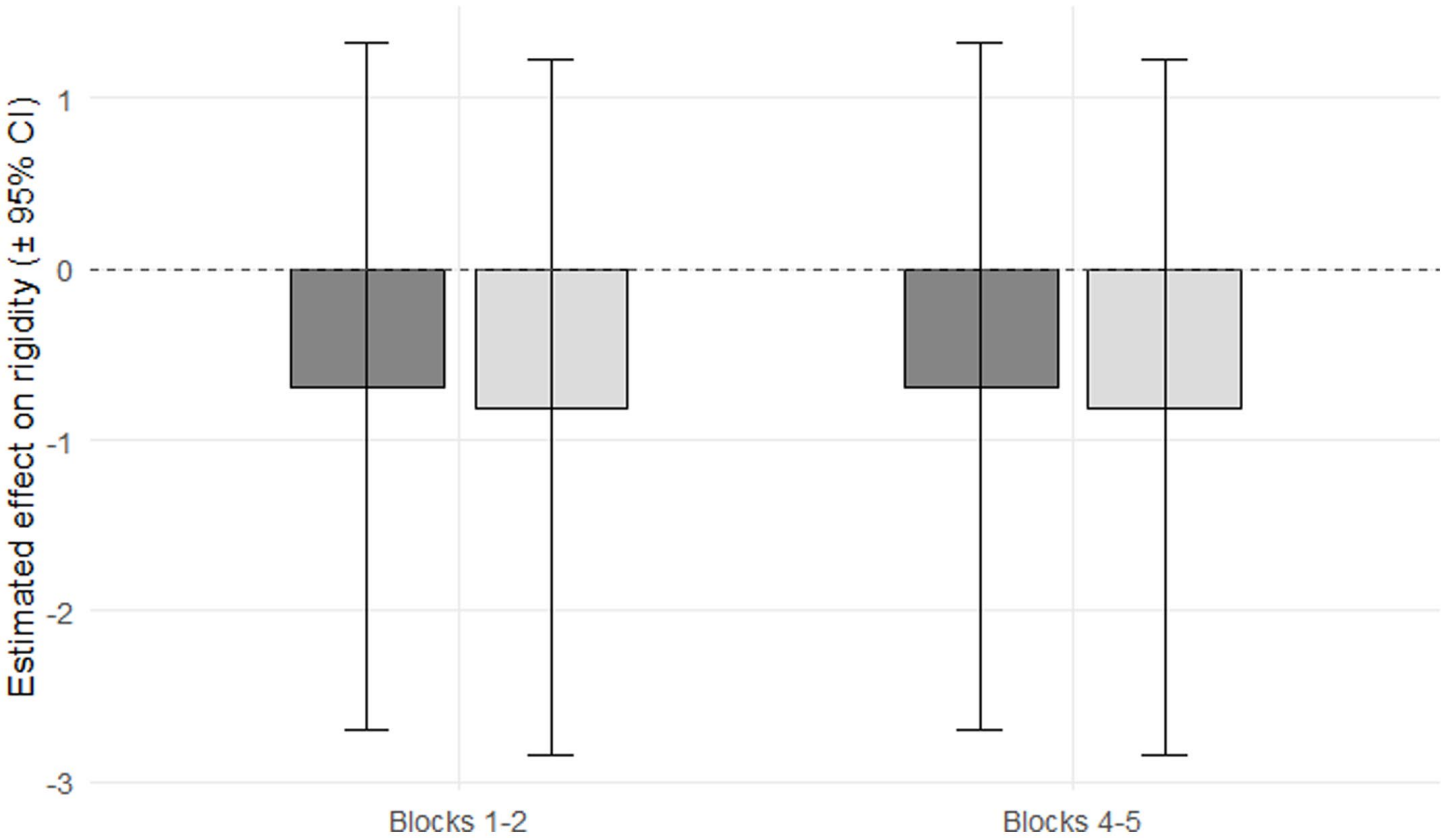
Effect of non-nutritive sweeteners (NNS; dark gray) and free sugars (light gray) on rigidity scores during early (Blocks 1–2) and late (Blocks 4–5) IGT periods. Neither substance had a significant effect on rigidity scores in either period (NNS: *p* = 0.499; free sugars: *p* = 0.433). Error bars represent ± 95% CI (confidence interval).

Rigidity score increased significantly from blocks 1–2 to blocks 4–5 (*p* < 0.0001). Daily NNS and free sugars intake were not significantly associated with rigidity scores (*p* = 0.499 and *p* = 0.433, respectively), and comparisons of the slopes for NNS vs. free sugars within each block revealed no significant difference: in blocks 1–2, the difference between slopes was 0.783 (SE = 1.01, *p* = 0.439), and in blocks 4–5, the difference was −0.654 (SE = 1.01, *p* = 0.517; Figure 2).

Women exhibited lower rigidity scores compared to men, regardless of the block period (*p* = 0.029). However, within each block period (1–2 or 4–5) men did not exhibit a significantly higher rigidity score compared to women (*p* = 0.175, Bonferroni-corrected; Supplementary Figures S2A,B).

Age had no significant effect on rigidity scores (*p* = 0.953).

#### Mean response time

Across the 100 trials, mean response times were significantly faster for option B compared to options A (−10.2%), C (−6.9%), and D (−7.6%; all *p* < 0.0001, Bonferroni-corrected). Option C was significantly faster than option A (−3.0%, *p* = 0.026, Bonferroni-corrected). No significant differences were observed between options D and A (*p* = 0.173, Bonferroni-corrected) or between options D and C (*p* = 1.000, Bonferroni-corrected; Supplementary Figure S3).

Daily NNS and free sugars intake had no significant effect on mean response across the 100 trials (*p* = 0.496 and *p* = 0.238, respectively; Table 9). At the beginning of the task (the first 40 trials), free sugars tend to exhibit higher mean response times (Supplementary Table S1). Age was positively associated with higher mean response time across the 100 trials (*p* = 0.009; Table 9). Age effect was significant during the first 40 trials and gradually decreased over subsequent trials, becoming non-significant by Block 5 (Supplementary Table S1). Women also exhibited longer mean response times compared to men (+17.6%; *p* = 0.002, Bonferroni-corrected; Table 9).

**Table 9:** Effect of predictors on mean response time across all 100 trials.

Predictor	β (SE) ^1^	Student test	*p*-value
Sweeteners (log-standardized)	0.02 (0.03)	0.68	0.496
Free sugars (log-standardized)	0.03 (0.03)	1.18	0.238
Age (standardized)	0.07 (0.03)	2.61	0.009**
Gender (Women vs. Men)	0.16 (0.05)	3.15	0.002**

1 β, coefficient; SE, Standard Error.

Bold values and “*” indicate statistically significant differences (*p* < 0.05).

Beyond these descriptive and predictive effects, we examined whether decision quality (proportion of advantageous choices, C/D) was related to mean response time. Correlations were generally non-significant (*p* = 0.853). In blocks 1–4, correlation coefficients were close to zero (*r* = −0.033 to 0.088, all *p* > 0.05). In block 5, a small but significant negative correlation emerged (*r* = −0.148, *p* = 0.013).

### Game of dice task

#### Probability of selecting a combination of dice faces

**Figure 3:**
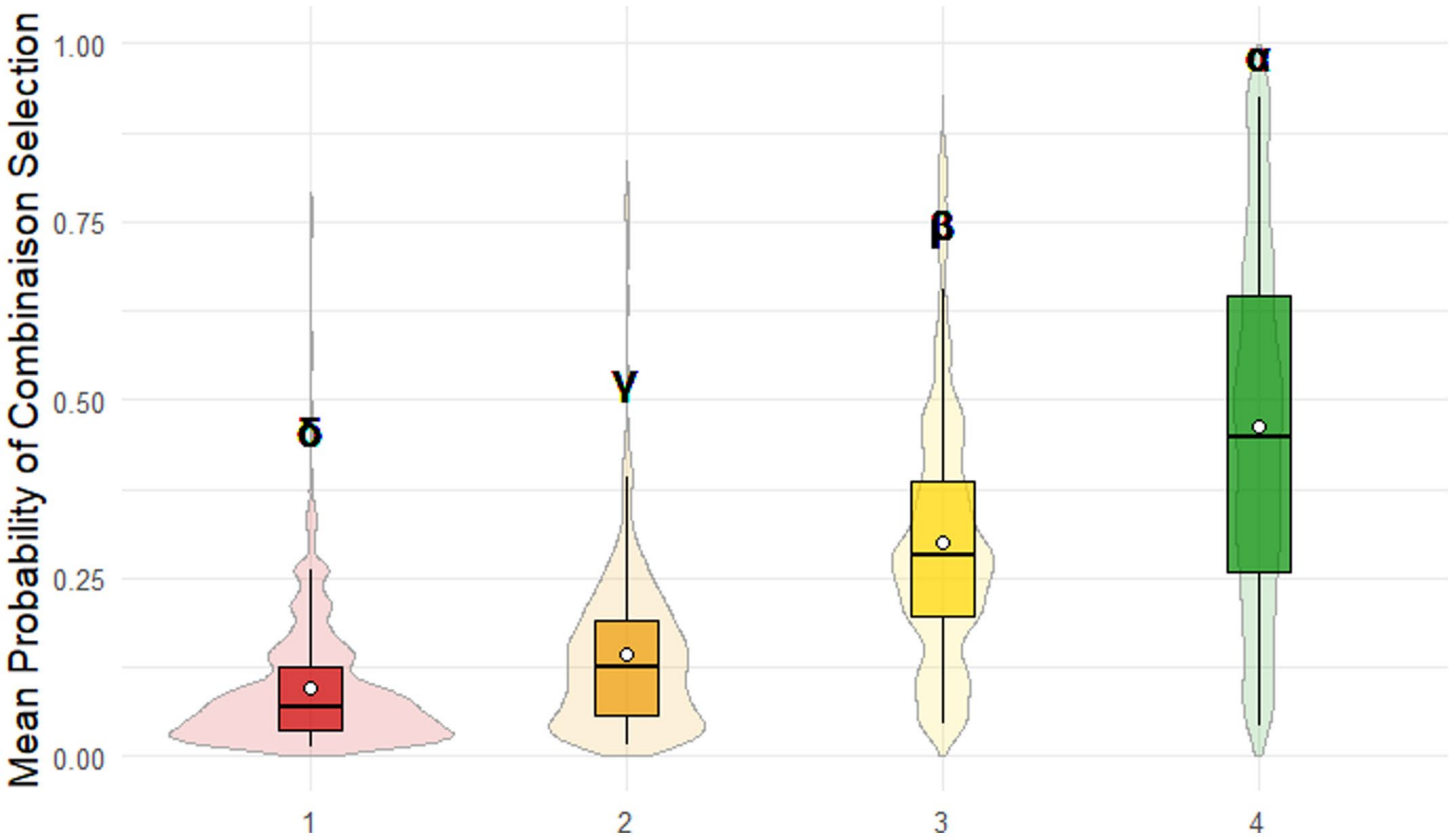
Game of Dice Task (GDT) performance. The mean probabilities of selecting each combination followed the descending order: 4-face > 3-face > 2-face > 1-face. Combinations are color-coded as follows: 1-face (red), 2-face (orange), 3-face (yellow), 4-face (green). Different Greek letters indicate significant differences between options (*p* < 0.05).

The probability of selecting a specific dice face combination differed significantly across the 18 trials. The probability of selecting a 4-face combination was significantly the highest (p(4 faces) = 0.46 ± 0.25; *p* < 10^−5^), while the probability of selecting a 1-face combination was significantly the lowest (p(1 face) = 0.10 ± 0.09; *p* < 10^−4^). The probability of selecting a 2-face combination (p(2 faces) = 0.14 ± 0.11) was significantly lower than the probability of selecting a 3-face combination (p(3 faces) = 0.30 ± 0.17; *p* < 10^−15^; Figure 3).

Result 2. Daily NNS intake was significantly and positively associated with selecting a 1-face combination over a 2-face combination (OR = 1.35, 95% CI [1.12–1.62]; *p* = 0.006, Bonferroni-corrected; Table 10). A trend toward a positive association between NNS intake and the probability of selecting 1-face combination over a 3-face combination was also observed (OR = 1.27, 95% CI [1.05–1.54]; *p* = 0.055, Bonferroni-corrected; Table 10). In contrast, daily free sugars intake had no significant effect on the probability of selecting any combination (Table 10).

**Table 10:** Effect of predictors on combination selection across all 18 trials of the GDT.

Predictor: 1 vs. 4^1^	OR [95% CI]^2^	*p*-value	*p*-value Bonferroni^3^
Sweeteners (log-standardized)	1.12 [0.88, 1.44]	0.344	1.000
Free sugars (log-standardized)	1.07 [0.85, 1.36]	0.553	1.000
Age (standardized)	1.32 [1.05, 1.66]	0.018*	0.073
Gender (Women vs. Men)	1.35 [0.88, 2.07]	0.171	0.684
Predictor: 2 vs. 4			
Sweeteners (log-standardized)	0.85 [0.67, 1.08]	0.173	0.691
Free sugars (log-standardized)	1.04 [0.82, 1.33]	0.725	1.000
Age (standardized)	1.36 [1.08, 1.72]	<0.01**	0.040*
Gender (Women vs. Men)	1.64 [1.07, 2.53]	0.024*	0.096
Predictor: 3 vs. 4			
Sweeteners (log-standardized)	0.87 [0.70, 1.08]	0.210	0.841
Free sugars (log-standardized)	1.05 [0.84, 1.30]	0.674	1.000
Age (standardized)	1.18 [0.96, 1.45]	0.119	0.477
Gender (Women vs. Men)	1.61 [1.10, 2.36]	0.015*	0.060
Predictor: 1 vs. 3^1^			
Sweeteners (log-standardized)	1.27 [1.05, 1.54]	0.014*	0.055
Free sugars (log-standardized)	1.03 [0.86, 1.24]	0.742	1.000
Age (standardized)	1.12 [0.94, 1.34]	0.217	0.867
Gender (Women vs. Men)	0.87 [0.62, 1.22]	0.431	1.000
Predictor: 2 vs. 3			
Sweeteners (log-standardized)	0.96 [0.82, 1.14]	0.661	1.000
Free sugars (log-standardized)	1.01 [0.86, 1.19]	0.914	1.000
Age (standardized)	1.16 [0.99, 1.36]	0.064	0.257
Gender (Women vs. Men)	1.06 [0.79, 1.43]	0.695	1.000
Predictor: 1 vs. 2^1^			
Sweeteners (log-standardized)	1.35 [1.12, 1.62]	0.001**	0.006**
Free sugars (log-standardized)	1.04 [0.87, 1.24]	0.682	1.000
Age (standardized)	0.99 [0.83, 1.17]	0.864	1.000
Gender (Women vs. Men)	0.88 [0.64, 1.21]	0.432	1.000

1 Comparisons are made between each combination and its respective reference: 1, 2, and 3 vs. 4; 1 and 2 vs. 3; and 1 vs. 2, as indicated in the table.

2 Odds ratios (OR) are exponentiated coefficients from the multinomial logistic model adjusted for covariates. 95% CI, 95% confidence interval.

3 *p*-values were adjusted using the Bonferroni correction, applied separately within each equation and only to the primary predictors of interest: NNS intake, free sugars intake, age, and gender. Bold values and “*” indicate statistically significant differences (*p* < 0.05).

Age was slightly and positively associated with selecting a 2-face combination over a 4-face combination (OR = 1.36, 95% CI [1.08–1.72]; *p* = 0.040, Bonferroni-corrected; Table 10), with a similar trend observed for selecting a 1-face combination over a 4-face combination (OR = 1.32, 95% CI [1.05–1.66]; *p* = 0.073, Bonferroni-corrected; Table 10).

Women tended to select 2-face and 3-face combinations more often than the 4-face combination, and more frequently than men (OR = 1.64, 95% CI [1.07–2.53]; *p* = 0.096; OR = 1.61, 95% CI [1.10–2.36]; *p* = 0.060, Bonferroni-corrected; Table 10).

#### Mean response time

Mean response times were significantly longer for 1-face and 2-face combinations compared to 3-face (+37.3% and +26.7%, respectively; both *p* < 0.0001; Bonferroni-corrected) and 4-face combinations (+90.5% and +75.7%, respectively; both *p* < 0.0001, Bonferroni-corrected). Response times were also significantly longer for 3-face compared to 4-face combinations (+38.7%, *p* < 0.0001, Bonferroni-corrected). No significant difference was observed between 1-face and 2-face combinations (*p* = 0.747, Bonferroni-corrected; Supplementary Figure S5).

Daily intake of NNS (*p* = 0.775), free sugars (*p* = 0.412), age (*p* = 0.344) had no significant effect on mean response times. Women exhibited longer mean response times compared to men (+30.9%; *p* < 0.0001; Supplementary Table S2).

### Binary dictator game

#### Probability of making altruistic choices: distributions of tokens

**Figure 4:**
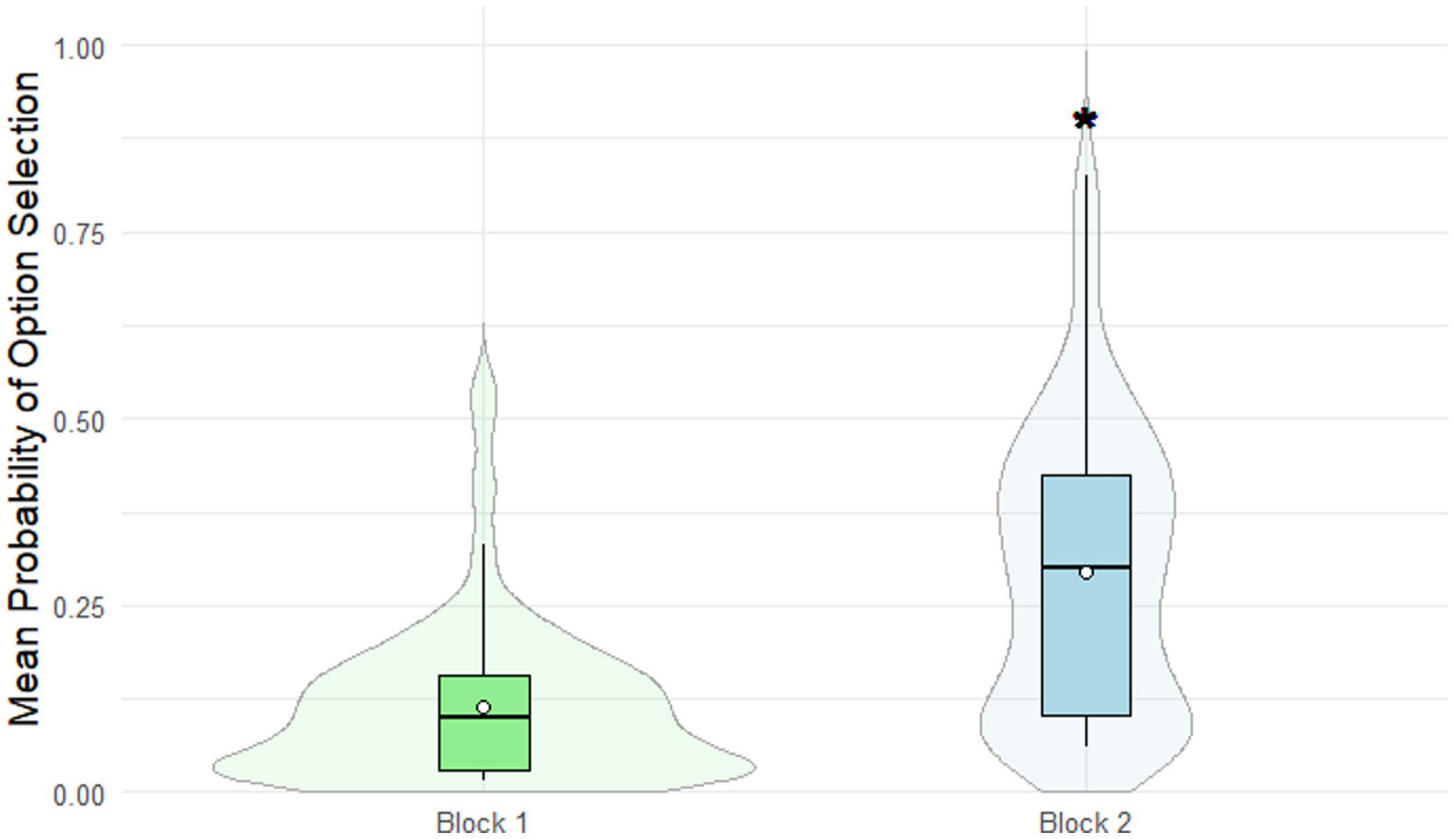
Binary dictator game performance. Altruistic choices were more frequently selected in the “unequal/unequal” (Block 2) condition compared to the “equal/unequal” (Block 1) condition (*p* < 0.0001, Bonferroni-corrected). Gain distribution types are color-coded as follows: equal/inequal distribution (light green), inequal/inequal distribution (light blue). *indicates a significant difference (*p* < 0.05).

The probability of selecting an altruistic choice varied significantly depending on the type of gain distribution. Participants were significantly more likely to select an altruistic choice in the “unequal/unequal” condition (0.30 ± 0.19) compared to the “equal/unequal” condition (0.11 ± 0.11; *p* < 0.0001, Bonferroni-corrected, Figure 4).

Result 3. Daily NNS and free sugars intake had no significant effect on the probability of selecting an altruistic choice, regardless of the gain distribution type (OR = 0.90, 95% CI [0.76–1.07]; *p* = 0.946; OR = 1.03, 95% CI [0.87–1.22]; *p* = 1.000, Bonferroni-corrected; Supplementary Table S4). Age was significantly and positively associated with selecting an altruistic choice, regardless of the gain distribution type (OR = 1.35, 95% CI [1.14–1.60]; *p* = 0.002) and within each distribution type (OR = 1.37, 95% CI [1.18–1.60]; *p* < 0.0001; OR = 1.23, 95% CI [1.06–1.42]; *p* = 0.021, Bonferroni-corrected; Table 11).

**Table 11:** Effect of predictors on the probability of selecting the altruistic option, in the “equal/unequal” or “unequal /unequal” condition.

Predictor: “equal/unequal”	OR [95% CI] ^1^	p-value	P-value Bonferroni ^2^
Sweeteners (log-standardized)	0.94 [0.80, 1.10]	0.432	1.000
Free sugars (log-standardized)	1.15 [0.98, 1.36]	0.094	0.377
Age (standardized)	1.37 [1.18, 1.60]	<0.0001***	<0.0001***
Gender (Women vs. Men)	1.05 [0.78, 1.41]	0.752	1.000
Predictor: “unequal /unequal”			
Sweeteners (log-standardized)	0.91 [0.78, 1.05]	0.196	0.783
Free sugars (log-standardized)	0.97 [0.83, 1.13]	0.687	1.000
Age (standardized)	1.23 [1.06, 1.42]	0.005**	0.021*
Gender (Women vs. Men)	1.46 [1.11, 1.92]	0.007**	0.028*

1 Odds ratios are exponentiated coefficients from the multinomial logistic model adjusted for covariates. 95% CI, 95% confidence interval.

2 *p*-values were adjusted using the Bonferroni correction, applied only to the primary predictors of interest: NNS intake, free sugars intake, age, and gender. Bold values and “*” indicate statistically significant differences (*p* < 0.05).

Women tented to be more likely than men to select altruistic choices across all distribution types (OR = 1.41, 95% CI [1.03–1.92]; *p* = 0.120, Bonferroni-corrected; Supplementary Table S4), with a significant effect observed in the “unequal/unequal” condition (OR = 1.46, 95% CI [1.11–1.92]; *p* = 0.028, Bonferroni-corrected; Table 11).

#### Mean response time

Mean response times for altruistic choices did not significantly differ between the “equal/unequal” and “unequal/unequal” conditions (*p* = 0.473, Bonferroni-corrected; Supplementary Figure S6).

NNS (*p* = 0.838), age (*p* = 0.397) and gender (*p* = 0.358) had no significant effect on mean response times, regardless of the type of gain distribution. Daily free sugars intake tended to be negatively associated with mean response times (*p* = 0.010; Supplementary Table S3).

### Prisoner’s dilemma

#### Probability of making cooperative choices: 3 different scenarios

**Figure 5:**
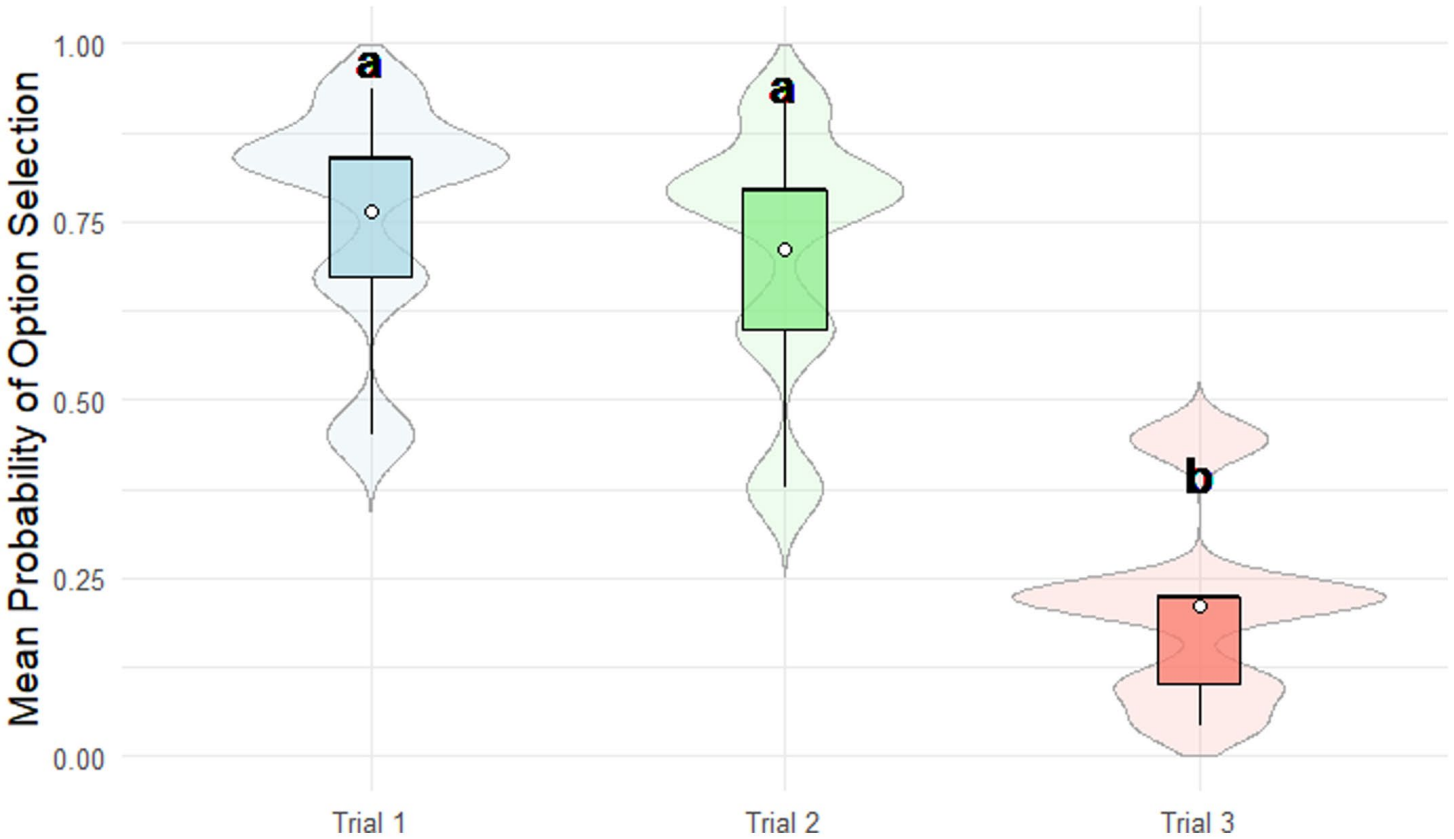
Prisoner’s dilemma performance. Cooperative choices were significantly less frequent in Trial 3 (partner’s hypothetical betrayal) compared to Trials 1 (deliberate choice) and 2 (partner’s hypothetical cooperation; *p* < 0.0001, Bonferroni-corrected). Different Greek letters indicate significant differences between options (*p* < 0.05). Scenario types are color-coded as follows: deliberate choice (light blue), partner’s hypothetical cooperation (light green), partner’s hypothetical betrayal (light coral). Different Greek letters indicate significant differences between options (*p* < 0.05).

The probability of selecting a cooperative -choice differed significantly across the three experimental conditions: Trial 1 (deliberate choice), Trial 2 (partner’s hypothetical cooperation), and Trial 3 (partner’s hypothetical betrayal). The probability was significantly lowest in Trial 3 (0.21 ± 0.13; *p* < 0.0001, Bonferroni-corrected), while no significant difference was observed between Trial 1 (0.76 ± 0.16) and Trial 2 (0.71 ± 0.17; *p* = 0.423, Bonferroni-corrected; Figure 5).

Result 4. Daily intake of NNS (OR = 1.17, 95% CI [0.90–1.52]; *p* = 0.993, Bonferroni-corrected), free sugars intake (OR = 0.97, 95% CI [0.74–1.26]; *p* = 1.000, Bonferroni-corrected), age (OR = 1.18, 95% CI [0.91–1.53]; *p* = 0.839, Bonferroni-corrected), and gender (OR = 1.12, 95% CI [0.70–1.80]; *p* = 1.000, Bonferroni-corrected) had no significant effect on the probability of selecting a cooperative choice, regardless of trial condition (Supplementary Table S6).

#### Mean response time

Mean response times for cooperative choices were significantly longer in Trial 2 compared to Trial 1 (+73.0%) and Trial 3 (+117.7%; both *p* < 0.0001, Bonferroni-corrected), while no significant difference was found between Trial 1 and Trial 3 (*p* = 0.438, Bonferroni-corrected; Supplementary Figure S7).

None of the explanatory variables of interest had a significant effect on mean response time (Supplementary Table S5).

### Emotion regulation questionnaire

Result 5. Daily NNS intake tended to be positively associated with cognitive reappraisal scores (*p* = 0.062) but had no significant effect on expressive suppression scores (*p* = 0.129). Daily free sugars intake was negatively associated with both emotion regulation scores (*p* = 0.027 and *p* = 0.028, respectively). Age tended to be associated with lower expressive suppression scores and had no significant effect on cognitive reappraisal scores (*p* = 0.056 and *p* = 0.438, respectively). Men exhibited significantly higher expressive suppression scores (3.99 ± 1.35) compared to women (3.29 ± 1.43; *p* < 0.0001). No significant gender differences were observed in cognitive reappraisal scores (*p* = 0.833; Tables 12, 13).

**Table 12:** Predictor’s effect on the cognitive reappraisal scores.

Predictor^1^	*β* (SE)^2^	Student test	*p*-value
Sweeteners (log-standardized)	0,13 (0,07)	1.87	0.062
Free sugars (log-standardized)	−0.15 (0.07)	−2.22	0.027*
Age (standardized)	0.05 (0.07)	0.78	0.438
Gender (Women vs. Men)	−0.03 (0.12)	−0.21	0.833

1 Effects refer to cognitive reappraisal scores.

2 β, coefficient; SE, Standard Error.

Bold values and “*” indicate statistically significant differences (*p* < 0.05).

**Table 13:** Predictor’s effect on the expressive suppression scores.

Predictor^1^	β (SE)^2^	Student test	*p*-value
Sweeteners (log-standardized)	0.14 (0.09)	1.52	0.129
Free sugars (log-standardized)	−0.21 (0.10)	−2.21	0.028*
Age (standardized)	−0.18 (0.09)	−1.92	0.056
Gender (Women vs. Men)	−0.74 (0.17)	−4.33	<0.0001***

1 Effects refer to expressive suppression scores.

2 β, coefficient; SE, Standard Error.

Bold values and “*” indicate statistically significant differences (*p* < 0.05).

## Discussion

This study examined the long-term associations of NNS intake with cognitive performance and socio-emotional behaviors in healthy French adults (18–30 years). Only one hypothesis was supported: NNS intake tended to be positively associated with the probability of selecting an advantageous yet risky option relative to its non-risky counterpart under experiential risk (IGT) and with selecting the riskiest option under explicit risk (GDT). Exploratory analyses indicated that higher free sugars intake tended to be negatively associated with the probability of selecting a disadvantageous and non-risky option relative to its risky counterpart during the IGT but showed no consistent effect in the GDT. Additionally, higher free sugars intake was associated with lower emotion regulation scores, whereas NNS intake tended to be positively associated with cognitive reappraisal. Neither NNS nor sugar was associated with prosocial decisions (altruism or cooperation).

### IGT—NNS and free sugars effects on ambiguity & experiential risk

In the IGT, Option B (disadvantageous/ “risky”) was most frequently selected, while Option D (advantageous/ “risky”) was least selected.

During the first 60 trials, daily NNS and free sugars intake were not significantly associated with the probability of selecting disadvantageous (A/B) or advantageous (C/D) options, nor with the selection of “risky” (B/D) vs. “non-risky” (A/C) alternatives. Associations emerged only in the later blocks.

Daily NNS intake tended to be positively associated with selecting Option D over Option C in the last 20 trials and across all trials, suggesting that NNS consumption tended to be associated with a greater selection of an advantageous yet risky option relative to its advantageous non-risky counterpart during the later experiential risk-taking phase. This pattern is consistent with Hamelin et al. (20), who reported that adult mice exposed to a 0.1% saccharin solution exhibited a significant increase in advantageous choices compared to controls or groups receiving 1% sucrose or 0.012% saccharin solutions during the experiential risk-taking phase. Increased risk-taking was also observed in the 0.012% saccharin group compared to controls.

Daily free sugars intake showed a progressive negative association with selecting Option A over B in Block 4, becoming significant in Block 5, with a similar trend observed across all trials. Higher free sugars consumption tended to be associated with lower selection of a disadvantageous and non-risky option relative to its disadvantageous and risky counterpart. Similarly, Hamelin et al. (20) reported that prolonged consumption of a 1% sucrose solution increased risk-taking in adult mice compared to controls under experiential risk.

However, free sugars intake also tended to be positively associated with selecting advantageous options (C and D) over option A during the last 20 trials. This result contrasts from Laugero and Keim (52), who linked sugar to disadvantageous choices.

In addition, neither NNS nor free sugars intake was associated with rigidity scores. Direct comparisons of NNS vs. sugar within each block revealed no significant differences, indicating that NNS not differ from free sugars in its correlation with rigidity over time. These finding contrasts with Hamelin et al. (20), in which mice consuming saccharin or sucralose exhibited greater behavioral rigidity compared with controls or sucrose-treated groups.

Finally, decision quality (advantageous C/D choices) was negatively correlated with mean response time during the last 20 trials, consistent with learning and strategy adoption.

### GDT—NNS and free sugars effects on explicit risk

A significant positive association was observed between the risk level of a dice combination and its selection probability. The most cautious and advantageous combination (4-face combination) was selected most frequently, whereas the riskiest and most disadvantageous combination (1-face combination) was the least frequently chosen.

Daily NNS intake was significantly and positively associated with selecting the riskiest 1-face combination over the moderately risky 2-face combination, with a similar positive trend observed for selecting the 1-face combination over the moderately safe 3-face combination. In contrast, daily free sugars consumption was not significantly associated with the probability of selecting any combination under explicit risk. Therefore, NNS was associated with both experiential and explicit risk.

Interestingly, neither NNS nor free sugars intake was associated with mean response time. No significant differences were found between risky combinations (1-face vs. 2-face). The shortest mean response time was for 4-face combination, suggesting greater risk requires longer cognitive processing.

Although we did not measure neural activity, meta-analyses suggest that risky decision-making engages a distributed neural network, notably the anterior insula (risk/ambiguity processing), the anterior cingulate cortex (ACC), and caudate (reward/motivation/affective processes) (18, 19, 53–55). The “risk matrix” model (56) posits that anterior insula activation reflects aversive outcome anticipation (e.g., monetary loss), with ACC modulating insula activity to shape risk behavior. Additional frontal regions—the orbitofrontal cortex (OFC), the vmPFC, and the pre-supplementary motor area (pre-SMA)—are also implicated (52, 57, 58). Future studies should examine whether habitual NNS or sugar consumption modulates these circuits during decision-making under experiential and explicit risk.

### Binary dictator game—NNS and free sugars effects on altruism

The probability of selecting an altruistic choice was significantly higher in “unequal/unequal” contexts. Daily NNS and free sugars intake were not associated with the probability of selecting an altruistic choice, regardless of distribution type.

While NNS intake was not associated to mean response times, higher free sugars intake tended to be associated with shorter response times across gain distribution types, and mean response times did not significantly differ across conditions.

### Prisoner’s dilemma—NNS and free sugars effects on cooperation

Participants showed a significantly lower probability of selecting cooperative options in the “hypothetical partner betrayal” condition (Trial 3), with no significant differences between “deliberate choice” (Trial 1) and “hypothetical partner cooperation” (Trial 2) conditions. Neither NNS nor free sugars intake was associated with the probability of selecting a cooperation option or mean response times across conditions.

No significant differences were found between Trial 1 and Trial 3, while Trial 2 exhibited the longer mean response times.

No evidence was found for moderate-to-large effects of NNS or free sugars on prosocial behavior in humans. With a sample size of *n* = 291, the study had 80% power to detect odds ratios ≥ 1.50 or ≤ 0.67 for altruism, and ≥ 1.39 or ≤ 0.72 for cooperation. The absence of significant associations therefore suggests that moderate-to-large effects are unlikely, although subtle effects cannot be excluded. In contrast, an animal study shows a contrasting pattern: Choi et al. (3) reported that prolonged sucrose intake (8 weeks) increased both the frequency and total duration of aggressive behaviors in adult mice compared to controls and aspartame groups.

### ERQ—NNS and free sugars effects on emotion regulation

Daily NNS intake tended to be positively associated with cognitive reappraisal scores but had no significant effect on expressive suppression scores. In contrast, higher free sugars consumption was associated with lower cognitive reappraisal and expressive suppression scores. Further studies are needed to clarify the associations between NNS or free sugar intake and self-reported habitual use of distinct emotion regulation strategies in healthy young adults.

### Limitations and strengths

This study is limited by its cross-sectional design, which precludes causal conclusions, and by its homogeneous sample of healthy young adults, limiting generalizability. Moreover, the q-FFQ provides an estimate of NNS and free sugars intake rather than a precise quantification. Both social desirability and recall biases may have led some participants to under- or overestimate their consumption. In addition, free sugars intake, estimated from total sugars in foods and beverages, may had led to slight overestimation for dairy products because of intrinsic lactose content. Nevertheless, dairy-related items constituted a minority of the questionnaire, likely limiting the magnitude of this potential bias. In statistical models, certain potential confounding factors, such as caffeine or alcohol consumption and sleep patterns, were not considered and could have influenced the observed associations.

However, certain characteristics of the study contribute to its strenghts. The sample’s homogeneity is advantageous, as participants were selected during young adulthood, when the prefrontal cortex—critical for executive functions—reaches functional maturity (≈25 years), ensuring stable cognitive and emotional regulation (59). Repeating this study in younger or older populations with neuroimaging could clarify the influence of NNS or sugar on decision-making and brain maturation. Additional strengths include the use of a diverse battery of behavioral tasks, a comprehensive dietary assessment of French food products (over 450 items for NNS) at two independent time points, reducing random error and accounting for temporal or seasonal variation, and a relatively large sample size (*n* = 291), enhancing analytical robustness.

## Conclusion

This study investigated the relationship between daily NNS and free sugars consumption and decision-making, as well as socio-cognitive behavior, in healthy young adults. While neither dietary component was not associated with social functioning, they showed differential associations with risk-related cognitive and emotional processes. Specifically, daily NNS intake tended to be positively associated with selecting an advantageous yet risky option relative to its non-risky counterpart in the IGT (Block 5 and across all trials) and with selecting the riskiest option in the GDT, suggesting an association with risk-taking under both experiential and explicit risk. In contrast, higher free sugars intake showed a progressively negative association with the probability of selecting a disadvantageous non-risky option relative to its risky counterpart, emerging in Block 4 and reaching significance in Block 5, with a similar trend observed across all trials. No consistent associations were found with GDT performance.

Neither NNS nor free sugars intake had a significant effect on IGT rigidity scores or mean response times across all tasks (IGT, GDT, Binary Dictator Game, and Prisoner’s Dilemma).

In addition, free sugars intake tended to be negatively associated with emotion regulation scores (cognitive reappraisal and expressive suppression), whereas NNS intake tended to be positively associated with cognitive reappraisal only. Overall, these findings suggest that daily NNS and free sugars may be differentially associated with experiential and explicit risk-taking in feedback-based contexts, as well as with aspects of emotion regulation. Future experimental and longitudinal studies are essential to clarify underlying neurobiological mechanisms and assess the long-term cognitive and behavioral consequences of habitual NNS and free sugars consumption.

## Data Availability

The datasets presented in this study can be found in online repositories. The names of the repository/repositories and accession number(s) can be found at: https://osf.io/u8rwz/files/osfstorage.
