# Supplementary material for: Chronic non-nutritive sweetener and free sugars consumption alters decision-making and risk-taking in young healthy adults
Source: Front Nutr. 2026 Apr 7;13:1796516. doi: 10.3389/fnut.2026.1796516 (PMC13098397; doi:10.3389/fnut.2026.1796516)
Supplement: Supplementary file 1 [file Table_1.docx]

Supplementary results


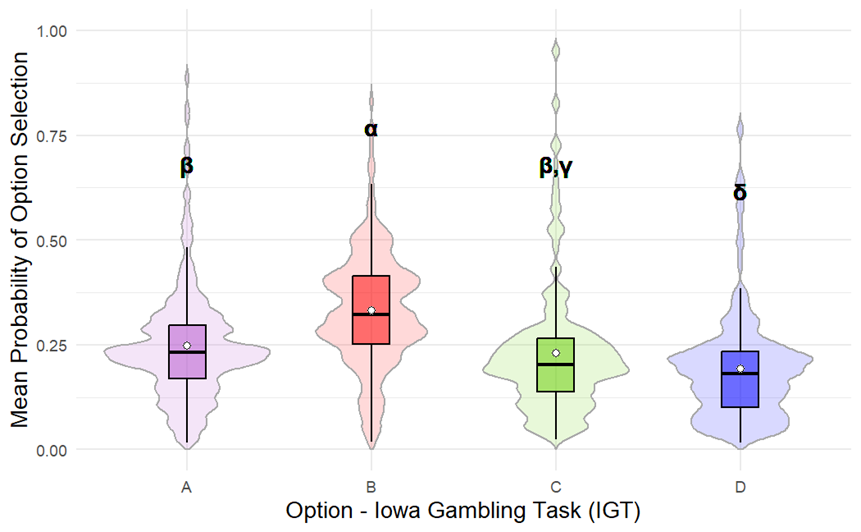


**Figure S1. Mean probability of option selection across all 100 IGT trials for all participants.** Across all 100 trials, mean selection probabilities decreased in the order B > A ≥ C > D. Options are color-coded as follows: A (purple), B (red), C (green), D (blue). Different Greek letters indicate significant differences between options (p < 0.05).

**(A)
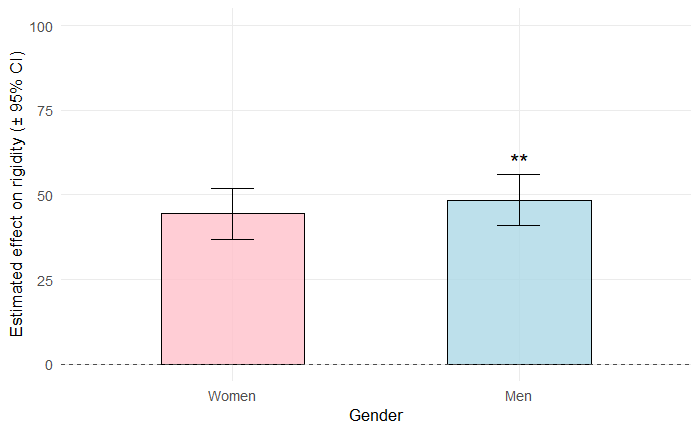
(B)
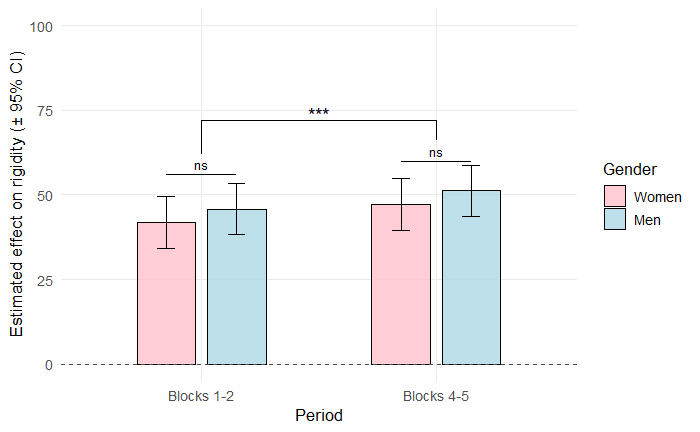
**

**Figure S2. Effect of gender on mean rigidity score. (A)** Women showed a significantly lower mean rigidity score than men, regardless of the block period (p = 0.029). **(B)** Within each block period (Blocks 1–2 or 4–5), men did not show a significantly higher rigidity score compared to women (p = 0.175, Bonferroni-corrected). Both sexes showed a significant increase in rigidity scores from Blocks 1–2 to Blocks 4–5 (p < 0.0001, Bonferroni-corrected). Men and women are represented in light blue and light pink, respectively. Error bars represent ± 95% CI (Confidence Interval).

**
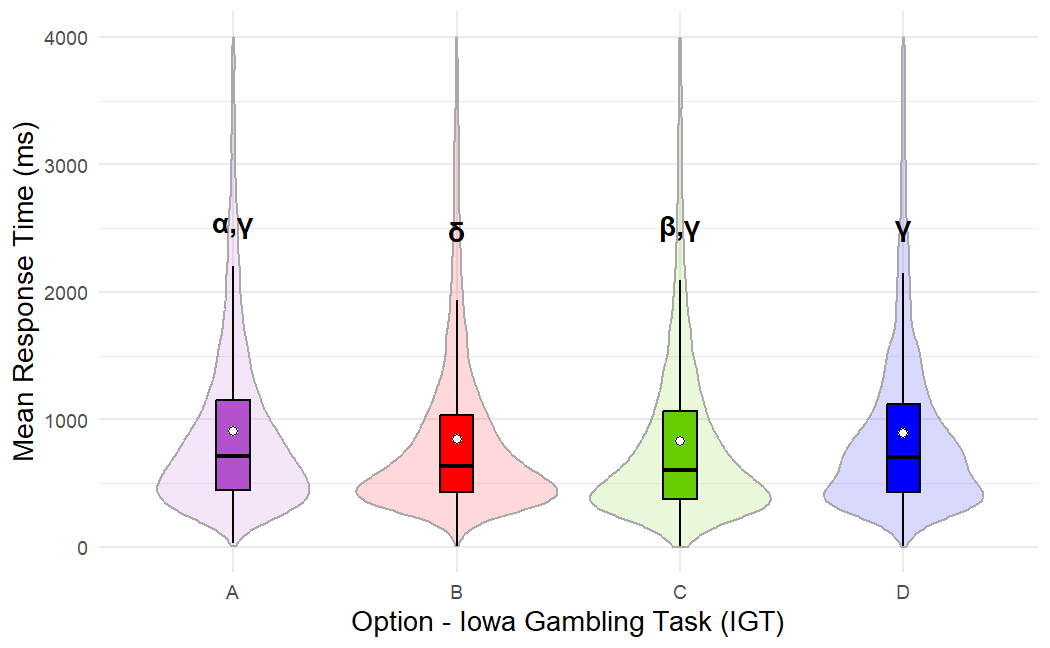
**

**Figure S3. Mean response time for IGT option selection for all participants.** Across the 100 trials, mean response times were significantly faster for option B compared to options A (−10.2%), C (−6.9%), and D (−7.6%) (all *p* < .0001, Bonferroni-corrected). Option C was significantly faster than option A (−3.0 %, *p* = 0.026, Bonferroni-corrected). No significant differences were observed between options D and A (*p* = 0.173, Bonferroni-corrected) or between options D and C (*p* = 1.000, Bonferroni-corrected). Options are color-coded as follows: A (purple), B (red), C (green), D (blue). Different Greek letters indicate significant differences between options (p < 0.05).

**
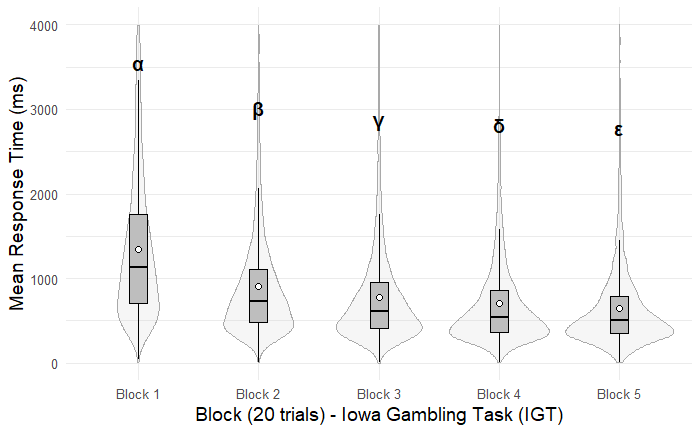
**

**Figure S4. Mean response times over 20-trial IGT blocks for all participants.** Mean response times were longest in Block 1 (all *p* < .0001, Bonferroni-corrected) and decreased progressively across subsequent blocks, reaching the shortest value in Block 5 (all pairwise comparisons *p* < .0001, Bonferroni-corrected). Different Greek letters indicate significant differences between options (p < 0.05).

**
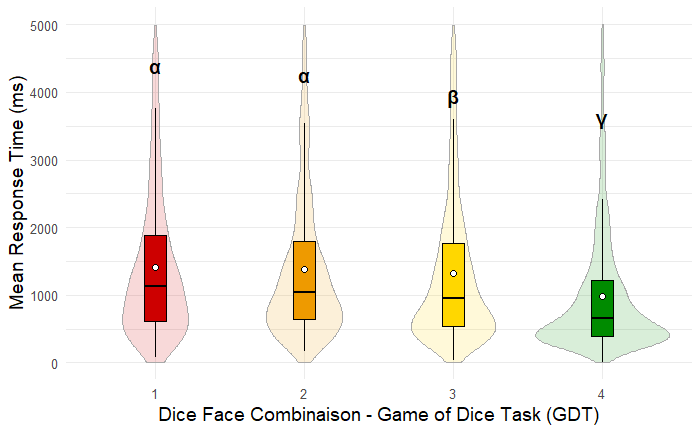
**

**Figure S5. Mean response time across GDT combination selections for all participants.** Mean response times were significantly longer for 1-face and 2-face combinations compared to 3-face (+37.3% and +26.7%, respectively; both *p* < .0001; Bonferroni-corrected) and 4-face combinations (+90.5% and +75.7%, respectively; both *p* < .0001, Bonferroni-corrected). Response times were also significantly longer for 3-face compared to 4-face combinations (+38.7%, p < .0001, Bonferroni-corrected). No significant difference was observed between 1-face and 2-face combinations (*p* = 0.747, Bonferroni-corrected). Combinations are color-coded as follows: 1-face (red), 2-face (orange), 3-face (yellow), 4-face (green). Different Greek letters indicate significant differences between options (p < 0.05).

**
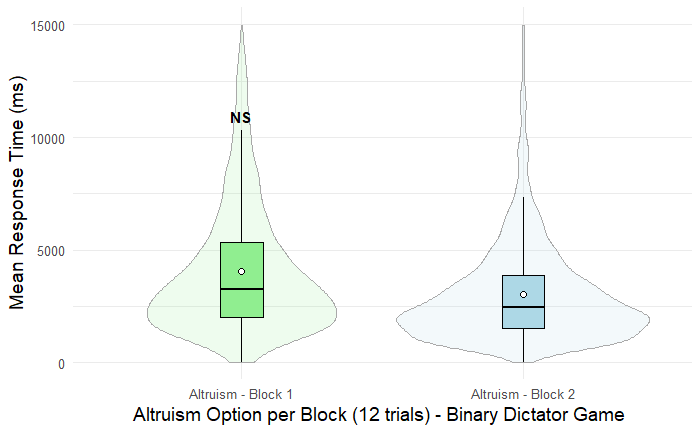
**

**Figure S6. Mean response time for altruistic option selections by gain distribution type for all participants.** There was no significant difference in mean response times between the "equal/unequal" and “unequal/unequal" conditions (*p* = 0.473, Bonferroni-corrected). Gain distribution types are color-coded as follows: equal/inequal distribution (light green), inequal/inequal distribution (light blue). NS indicates non-significant differences.

**
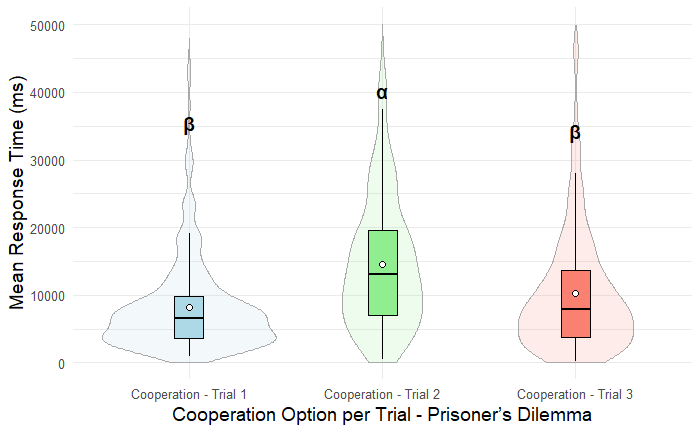
**

**Figure S7. Mean response time for cooperative option selections by type of scenario for all participants.** Mean response times were significantly longer in Trial 2 compared to Trial 1 (+73,0%) and Trial 3 (+117,7%) (both *p* < .0001, Bonferroni-corrected). No significant difference was observed between Trial 1 and Trial 3 (*p* = 0.438, Bonferroni-corrected). Different Greek letters indicate significant differences between options (p < 0.05). Scenario types are color-coded as follows: deliberate choice (light blue), partner’s hypothetical cooperation (light green), partner’s hypothetical betrayal (light coral). Different Greek letters indicate significant differences between options (p < 0.05).

**Table S1. Predictor’s effect on the mean response time within blocks of 20 trials in the IGT.**

| **Predictor: - Block 1** | **β (SE)** ^1^ | **Student test** | **P-value** |
| --- | --- | --- | --- |
| Sweeteners (log - standardized) | -0.013 (0.03) | -0.49 | 0.626 |
| Free sugars (log - standardized) | 0.06 (0.03) | 2.25 | **0.025 *** |
| Age (standardized) | 0.11 (0.03) | 4.17 | **<. 0001 ***** |
| Gender (Women vs Men) | 0.07 (0.05) | 1.45 | 0.147 |
| **Predictor: - Block 2** |  | | |
| Sweeteners (log - standardized) | 0.03 (0.03) | 0.85 | 0.394 |
| Free sugars (log - standardized) | 0.05 (0.03) | 1.72 | 0.085 **.** |
| Age (standardized) | 0.08 (0.03) | 2.86 | **0.004 **** |
| Gender (Women vs Men) | 0.19 (0.05) | 3.55 | **< 0.001 ***** |
| **Predictor: - Block 3** |  | | |
| Sweeteners (log - standardized) | 0.04 (0.04) | 1.00 | 0.315 |
| Free sugars (log - standardized) | 0.02 (0.04) | 0.43 | 0.664 |
| Age (standardized) | 0.06 (0.03) | 1.88 | 0.060 **.** |
| Gender (Women vs Men) | 0.17 (0.06) | 2.65 | **0.008 **** |
| **Predictor: - Block 4** |  | | |
| Sweeteners (log - standardized) | 0.03 (0.03) | 0.92 | 0.360 |
| Free sugars (log - standardized) | 0.02 (0.04) | 0.70 | 0.482 |
| Age (standardized) | 0.07 (0.03) | 1.93 | 0.054 **.** |
| Gender (Women vs Men) | 0.17 (0.06) | 2.68 | **0.007 **** |
| **Predictor: - Block 5** |  | | |
| Sweeteners (log - standardized) | 0.02 (0.04) | 0.55 | 0.584 |
| Free sugars (log - standardized) | 0.02 (0.04) | 0.40 | 0.692 |
| Age (standardized) | 0.04 (0.04) | 1.16 | 0.245 |
| Gender (Women vs Men) | 0.22 (0.07) | 3.21 | **0.001 **** |

^1^ β : coefficient ; SE : Standard Error.

*p* < 0.05: statistically significant.

**Table S2. Predictor’s effect on the mean response time in the GDT.**

| **Predictor:**  ^1^ | **β (SE)** ^2^ | **Student test** | **P-value** |
| --- | --- | --- | --- |
| Sweeteners (log - standardized) | 0.01 (0.04) | 0.29 | 0.775 |
| Free sugars (log - standardized) | 0.03 (0.04) | 0.82 | 0.412 |
| Age (standardized) | 0.03 (0.04) | 0.95 | 0.344 |
| Gender (Women vs Men) | 0.27 (0.07) | 4.10 | **<. 0001 ***** |

^1^ Effects refer to the mean response time in the GDT.

^2^ β : coefficient ; SE : Standard Error.

*p* < 0.05: statistically significant.

**Table S3. Predictor’s effect on the mean response time in the Binary Dictator Game.**

| **Predictor:**  ^1^ | **β (SE)** ^2^ | **Student test** | **P-value** |
| --- | --- | --- | --- |
| Sweeteners (log - standardized) | 0.01 (0.03) | 0.20 | 0.838 |
| Free sugars (log - standardized) | -0.05 (0.03) | -1.65 | 0.010 **.** |
| Age (standardized) | 0.02 (0.03) | 0.85 | 0.397 |
| Gender (Women vs Men) | 0.05 (0.05) | 0.92 | 0.358 |

^1^ Effects refer to the mean response time during 'total' altruistic choices in the Binary Dictator Game.

^2^ β : coefficient ; SE : Standard Error.

*p* < 0.05: statistically significant.

**Table S4. Predictor’s effect on total altruistic choice in the Binary Dictator Game.**

| **Predictor:** ^1^ | **OR [95% CI] ^2^** | **P-value** | **P-value Bonferroni ^3^** |
| --- | --- | --- | --- |
| Sweeteners (log - standardized) | 0.90 [0.76, 1.07] | 0.237 | 0.946 |
| Free sugars (log - standardized) | 1.03 [0.87, 1.22] | 0.735 | 1.000 |
| Age (standardized) | 1.35 [1.14, 1.60] | **< 0.001 ***** | **0.002 **** |
| Gender (Women vs Men) | 1.41 [1.03, 1.92] | **0.030 *** | 0.120 |

^1^ Effects refer to altruistic choice, regardless of the gain distribution type.

^2^ Odds ratios are exponentiated coefficients from the multinomial logistic model adjusted for covariates. 95% CI: 95% confidence interval.

^3^ P-values were adjusted using the Bonferroni correction, applied only to the primary predictors of interest: NNS intake, free sugars intake, age, and gender. *p* < 0.05: statistically significant.

**Table S5. Predictor’s effect on the mean response time in the Prisoner’s Dilemma.**

| **Predictor:**  ^1^ | **β (SE)** ^2^ | **Student test** | **P-value** |
| --- | --- | --- | --- |
| Sweeteners (log - standardized) | 0.05 (0.04) | 1.13 | 0.258 |
| Free sugars (log - standardized) | 0.001 (0.04) | 0.02 | 0.981 |
| Age (standardized) | 0.04 (0.04) | 0.92 | 0.360 |
| Gender (Women vs Men) | 0.08 (0.08) | 1.07 | 0.286 |

^1^ Effects refer to the mean response time during cooperative choices in the Prisoner’s Dilemma.

^2^ β : coefficient ; SE : Standard Error.

*p* < 0.05: statistically significant.

**Table S6**. **Predictor’s effect on the probability of selecting the cooperative option,** **regardless of the scenario type.**

| **Predictor:** | **OR [95% CI] ^1^** | **P-value** | **P-value Bonferroni ^2^** |
| --- | --- | --- | --- |
| Sweeteners (log - standardized) | 1.17 [0.90, 1.52] | 0.248 | 0.993 |
| Free sugars (log - standardized) | 0.97 [0.74, 1.26] | 0.797 | 1.000 |
| Age (standardized) | 1.18 [0.91, 1.53] | 0.210 | 0.839 |
| Gender (Women vs Men) | 1.12 [0.70, 1.80] | 0.632 | 1.000 |

^1^ Odds ratios are exponentiated coefficients from the multinomial logistic model adjusted for covariates. 95% CI: 95% confidence interval.

^2^ P-values were adjusted using the Bonferroni correction, applied only to the primary predictors of interest: NNS intake, free sugars intake, age, and gender. *p* < 0.05: statistically significant.
